# MitoSNO inhibits mitochondrial hydrogen peroxide generation by α-ketoglutarate dehydrogenase

**DOI:** 10.1016/j.jbc.2025.108510

**Published:** 2025-04-16

**Authors:** Olivia Chalifoux, Samantha Sterman, Ben Faerman, Meijing Li, Stephanie Trezza, Marek Michalak, Luis B. Agellon, Ryan J. Mailloux

**Affiliations:** 1 School of Human Nutrition, McGill University, Sainte-Anne-de-Bellevue, Quebec, Canada; 2 Department of Biochemistry, University of Alberta, Edmonton, Alberta, Canada

**Keywords:** alpha-ketoglutarate dehydrogenase, hydrogen peroxide, mitochondria, mitochondria-targeted therapy, nitrosation, oxidative distress

## Abstract

Here, we demonstrate mitochondrial hydrogen peroxide (mtH_2_O_2_) production by α-ketoglutarate dehydrogenase (KGDH) can be inhibited by mitochondria-targeted S-nitrosating agent (MitoSNO), alleviating lipotoxicity. MitoSNO in the nanomolar range inhibits mtH_2_O_2_ by ∼50% in isolated liver mitochondria without disrupting respiration, whereas the mitochondria-selective derivative used to synthesize MitoSNO, mitochondria-selective N-acetyl-penicillamine, had no effect on either mtH_2_O_2_ generation or oxidative phosphorylation. Additionally, mtH_2_O_2_ generation in isolated liver mitochondria was almost abolished when MitoSNO was administered in the low micromolar range. The potent inhibitory effect of MitoSNO was comparable to 2-keto-3-methyl-valeric acid and valproic acid, selective inhibitors for KGDH-mediated mtH_2_O_2_ production. S1QEL 1.1 (S1) and S3QEL (S3), which are known to selectively suppress mtH_2_O_2_ genesis through inhibition of complex I and complex III, respectively, without disrupting respiration, had little to no effect on mtH_2_O_2_ production by liver mitochondria. The MitoSNO also suppressed mtH_2_O_2_ production and partially rescued mitochondrial respiration in Huh-7 cells subjected to palmitate- and fructose-induced lipotoxicity. MitoSNO also prevented cell death and abrogated intrahepatic lipid accumulation in these Huh-7 cells. MitoSNO nullified mtH_2_O_2_ overgeneration and partially rescued oxidative phosphorylation in liver mitochondria from mice fed a high-fat diet. Our findings demonstrate that MitoSNO interferes with mtH_2_O_2_ production through KGDH S-nitrosation and may be useful in alleviating nonalcoholic fatty liver disease.

Mitochondrial hydrogen peroxide (mtH_2_O_2_) can be beneficial or deleterious to liver health. Complexes I and III of the electron transport chain (ETC) are credited to be the main sources of mtH_2_O_2_ in hepatocytes. This is based on many studies that have used classic inhibitors like rotenone and myxothiazol/antimycin A/stigmatellin and/or gene knockouts to modulate mtH_2_O_2_ release by complex I and III. The main issue is the classical inhibitors and genetic manipulations used to define complex I and III as the main mtH_2_O_2_ sources abrogate electron flow through the ETC, which overestimates the rate of mtH_2_O_2_ production by the complexes. To overcome this, new inhibitors for evaluating mtH_2_O_2_ production by complex I and III that do not disrupt the ETC or oxidative phosphorylation (OxPhos) were identified. N-cyclohexyl-4-(4-nitrophe-noxy)benzenesulfonamide was identified as the first site specific suppressor of mtH_2_O_2_ biosynthesis by complex I that does not disrupt respiration (1). Ensuing large scale chemical screening studies led to the identification and characterization of the S1QEL (S1; pronounced “*cycle*”) and S3QEL (S3; pronounced “*sequel*”) compounds, which are high-affinity and site-specific inhibitors for mitochondrial reactive oxygen species (mtROS) leakage from complexes I and III, respectively, that also do not alter ETC function (2, 3). S1 and S3 induce a maximum inhibition of mtH_2_O_2_ genesis by complex I and complex III at a concentration range of 0.1 to 1 μM in seven different cell lines without interfering with OxPhos or cell growth (3, 4, 5). S1 and S3 compounds also only inhibit mtH_2_O_2_ biosynthesis by complex I and III by ∼10 to 20%, depending on the cultured cell type (4). S1 and S3 have been shown to bind to the ubiquinone binding site, although how it prevents mtROS production without disrupting ETC function has not been delineated (5, 6). Because mitochondria can account for ∼90% of the total cellular hydrogen peroxide (H_2_O_2_) production, this would suggest that in some cell types, mitochondrial enzymes other than complex I and III may serve as the highest capacity mtH_2_O_2_ sources.

It is now accepted mammalian mitochondria can contain up to 12 mtH_2_O_2_ sources, with most situated upstream from complex I and III (7). Of these sources, there is compelling evidence showing α-ketoglutarate dehydrogenase (KGDH), a tricarboxylic acid cycle enzyme, is a potent generator of mtH_2_O_2_ in brain, muscle, and liver cells (7, 8, 9, 10, 11, 12). Implementation of a substrate-inhibitor toolkit that poisons various parts of the tricarboxylic acid cycle and ETC to interrogate the rate of mtH_2_O_2_ production by these 12 individual sources revealed KGDH is a high-capacity site for production when liver mitochondria are fueled with pyruvate, lactate, amino acids, and fatty acylcarnitines (11, 13, 14). Identification of KGDH as a main mtH_2_O_2_ source in liver and muscle was achieved using 2-keto-3-methylvaleric acid (KMV), a competitive inhibitor for α-ketoglutarate binding to the E1 subunit, devimistat, otherwise known as CPI-613, a lipoate analog that is in clinical trial for cancer treatment, and succinyl-phosphonate, a potent site-specific inhibitor of the enzyme (9, 11, 15). Furthermore, the recent implementation of S1 and S3 molecules as part of this substrate-inhibitor toolkit revealed KGDH was a more potent mtH_2_O_2_ source in liver mitochondria metabolizing pyruvate or fatty acylcarnitines when compared to complexes I and III (13). This observation is consistent with a study that used a gene deletion for dihydrolipoylamide succinyl-transferase or dihydrolipoamide dehydrogenase to show KGDH is a major mtH_2_O_2_ source in brain mitochondria (16).

Our research group recently reported KGDH is a potent mtH_2_O_2_ generator during mitochondrial fatty acid oxidation (13). Further, overgeneration of mtH_2_O_2_ by KGDH induced oxidative distress in hepatocytes after dietary fat overload causing nonalcoholic fatty liver disease (NAFLD) in male mice, which could be mitigated through KGDH redox modification (17). Mitochondria-targeted S-nitrosating agent (MitoSNO) has emerged as an important molecule with high therapeutic potential because of its capacity to dynamically inhibit mtH_2_O_2_ generation by mitochondria (18, 19). This is achieved through the S-nitrosation of Cys^59^ in the ND3 subunit of complex I, which protects the myocardium from ischemia-reperfusion injury caused by reverse electron transport from succinate (18, 19). S-nitrosation also orchestrates the activity of KGDH and inhibits mtH_2_O_2_ production through modification of the vicinal lipoamide thiols in the E2 subunit (20, 21). In the present study, we reveal acute treatment of liver mitochondria with MitoSNO nullifies mtH_2_O_2_ generation through KGDH S-nitrosation. This MitoSNO treatment is not disruptive toward OxPhos and protects against lipotoxicity induced by palmitate (PA) and fructose (fruc) overload by mitigating mtH_2_O_2_ over generation.

## Results

### S1 and S3 compounds minimally affect the rate of mtH_2_O_2_ production by isolated liver mitochondria

**Figure 1:**
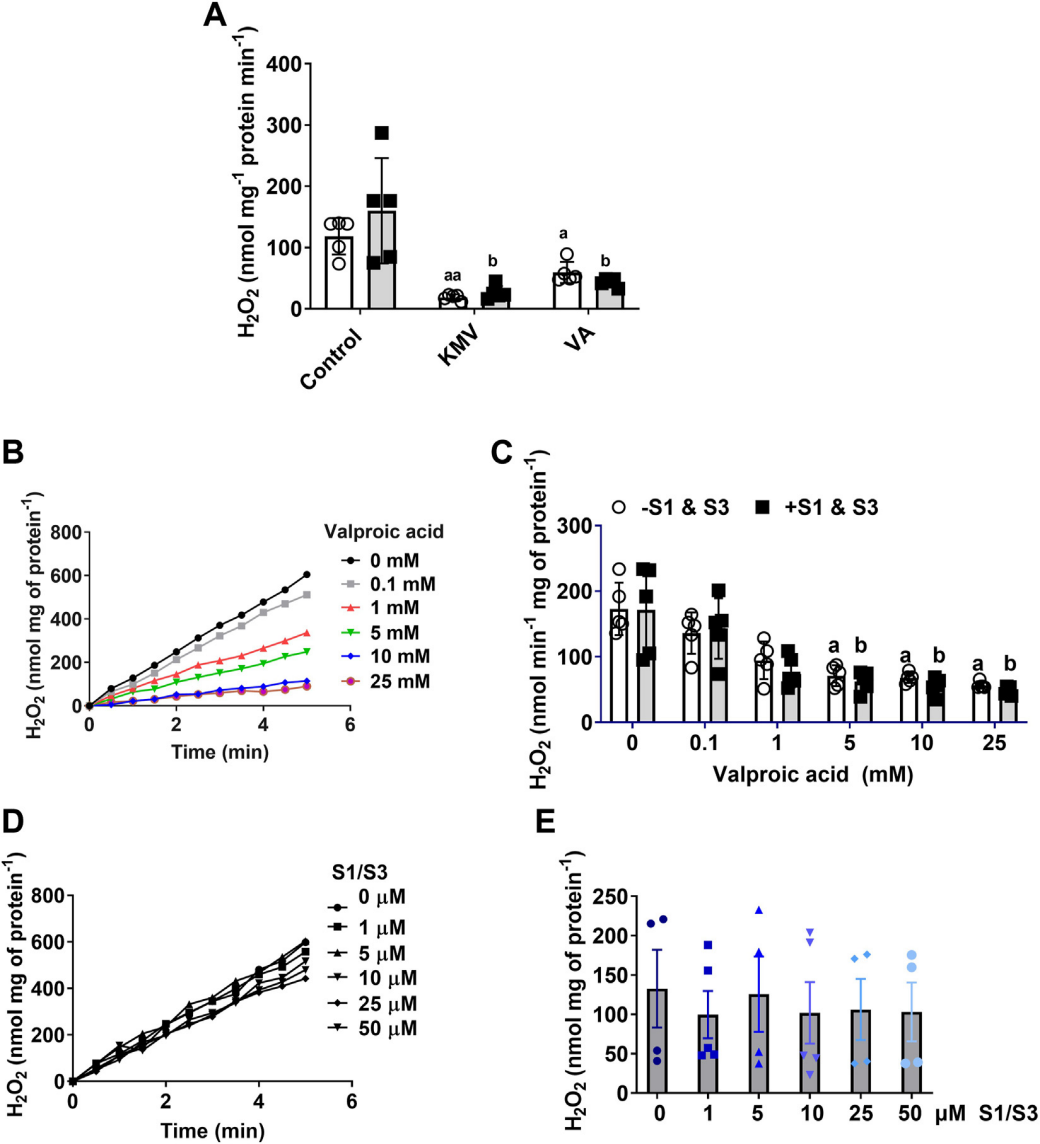
**KMV and valproic acid, inhibitors for KGDH, but not S1 or S3, are effective deactivators for mtH_2_O_2_ production in liver mitochondria fueled by pyruvate and malate.***A*, valproic acid is just as effective as KMV in the inhibition of mtH_2_O_2_ generation in the absence or presence of S1 and S3. Liver mitochondria fueled with pyruvate and malate were treated with or without 10 μM S1 and 10 μM S3. Some reactions were supplemented with KMV (10 mM) or valproic acid (10 mM) to test if KGDH was the main source of mtH_2_O_2_. For all panels: N = 5, mean ± SD, two-way ANOVA with a Tukey’s *post hoc* test. a = statistical comparisons in −S1 and S3 group. b = statistical comparisons in +S1 and S3 group. ◦ (*white bar*) = −S1 and S3 ▪ (*gray bar*) = +S1 and S3. *B*, trace data demonstrating the dose dependency for the valproic acid mediated inhibition of mtH_2_O_2_. This data were used to calculate the rate of mtH_2_O_2_ production in (*C*). N = 4, mean ± SD, one-way ANOVA with a Tukey’s *post hoc* test. a = the value is significantly different from the control (0 μM) in the −S1 and S3 group. b = the value is significantly different from the control (0 μM) in the +S1 and S3 group. ◦ (*white bar*) = −S1 and S3 ▪ (*gray bar*) = +S1 and S3. *D*, trace data demonstrating ETC inhibitors S1 and S3 do not inhibit mtH_2_O_2_ generation. This data were used to calculate the rate of mtH_2_O_2_ production in (*E*). N = 4, mean ± S.D., one-way ANOVA with a Tukey’s *post hoc* test. KGDH, α-ketoglutarate dehydrogenase; KMV, 2-keto-3-methylvaleric acid; mtH_2_O_2_, mitochondrial hydrogen peroxide.

First, we tested the effect of 10 μM S1 and 10 μM S3 with or without KMV (10 mM) and valproic acid (VA; 10 mM) on mtH_2_O_2_ production by liver mitochondria from mice. Figure 1*A* shows the combination of S1 and S3 had no significant effect on mtH_2_O_2_ production by liver mitochondria fueled with pyruvate and malate. Inclusion of KMV or VA, however, nearly abolished this mtH_2_O_2_ production (Fig. 1*A*). KMV is a well-documented competitive inhibitor of the E1 subunit of KGDH that almost abolishes mtH_2_O_2_ production by the enzyme complex (9, 22). However, whether VA, another KGDH inhibitor, can also nullify mtH_2_O_2_ production has never been tested (23). Figure 1, *B* and *C* shows the titration of VA from 0.1 mM to 25 mM into reaction chambers dose dependently decreases the rate of mtH_2_O_2_ production by liver mitochondria-oxidizing pyruvate and malate. Maximum inhibition of mtH_2_O_2_ production was achieved at 10 mM VA (Fig. 1, *B* and *C*). Furthermore, Figure 1*C* illustrates that including S1 and S3 at 10 μM each in the reaction chambers with VA did not significantly impact the rate of mtH_2_O_2_ production. Next, we sought to test the impact of increasing doses of S1 and S3 on the rate of mtH_2_O_2_ production by these liver mitochondria. Previous work has screened several S1 and S3 analogs, which all have been shown to selectively interfere with mtH_2_O_2_ production by complex I and complex III, respectively (3). Trace data in Figure 1*D* shows S1 and S3 did not impact mtH_2_O_2_ production, even when administered at doses as high as 50 μM. We also calculated the rate of mtH_2_O_2_ using this data. Figure 1*E* shows the combination of S1 and S3 did not significantly alter the rate of mtH_2_O_2_ generation, even when both compounds were given at 50 μM each. We also tested the impact of S1 and S3 on the rate of mtH_2_O_2_ production by mouse cardiac mitochondria (Fig. S1). When administered together, the S1 and S3 did not affect mtH_2_O_2_ generation in liver mitochondria but did abrogate production in cardiac mitochondria (Fig. S1). Figure S1 demonstrates administering 10 μM S1 and S3 in combination slightly increases state 3 respiration in liver mitochondria but does not alter state 4 or state 4_O_ oxygen consumption rate (OCR). In addition, the S1 and S3 compounds did not impact state 3, state 4, or state 4_O_ respiration in cardiac mitochondria (Fig. S1).

**Figure 2:**
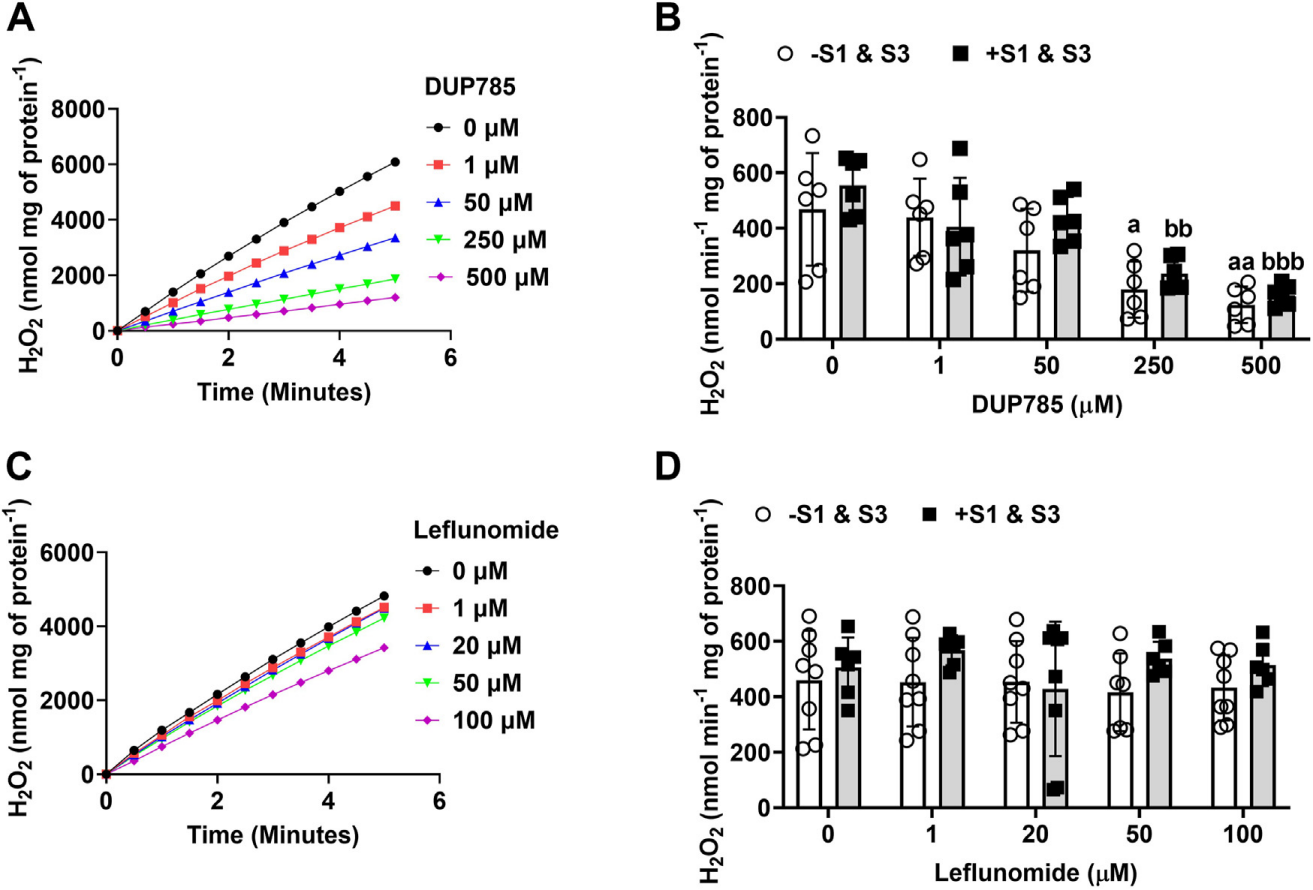
**DHODH is an important source of mtH_2_O_2_ in liver mitochondria.** Isolated liver mitochondria were treated with increasing amounts of DUP785 or leflunomide with or without S1 (10 μM) and S3 (10 μM) to test the impact of the DHODH and ETC inhibitors on mtH_2_O_2_ production. *A*, trace data demonstrating the dose dependency for the DUP785 mediated inhibition of mtH_2_O_2_. *B*, impact of increasing doses of DUP785 on the rate of mtH_2_O_2_ production by liver mitochondria treated with or without S1 (10 μM) and S3 (10 μM). *C*, trace data demonstrating the dose dependency for the leflunomide mediated inhibition of mtH_2_O_2_. *D*, impact of increasing doses of leflunomide on the rate of mtH_2_O_2_ production by liver mitochondria treated with or without S1 (10 μM) and S3 (10 μM). N = 6, mean ± SD, two-way ANOVA with a Tukey’s *post hoc* test. a = the value is significantly different from the control (0 μM) in the −S1 and S3 group. b = the value is significantly different from the control (0 μM) in the +S1 and S3 group. ◦ (*white bar*) = −S1 and S3 ▪ (*gray bar*) = +S1 and S3. DHODH, dihydroorotate dehydrogenase; mtH_2_O_2_, mitochondrial hydrogen peroxide.

Next, we tested the impact of S1 and S3 on mtH_2_O_2_ production using liver mitochondria energized with dihydroorotate, the substrate for dihydroorotate dehydrogenase (DHODH). DHODH catalyzes the rate-limiting step of pyrimidine biosynthesis by transferring electrons from dihydroorotate to the ETC, making this pathway a key driver of mtH_2_O_2_ production by complex I and complex III (24). DUP785 (Brequinar) and leflunomide have been shown to be potent anticancer and arthritis treatment agents that interfere with DHODH by binding to the narrow region in the hydrophobic tunnel motif of the enzyme that leads to the flavin mononucleotide-ubiquinone redox site (25). DUP785 titration assays revealed a progressive, dose-dependent inhibition of mtH_2_O_2_ production with increasing concentrations when liver mitochondria were fueled with dihydroorotate (Fig. 2*A*). Maximal inhibition of mtH_2_O_2_ production with the DUP785 was attained at 250 to 500 μM (Fig. 2*A*). Next, we interrogated the impact of the combined treatment of liver mitochondria S1 and S3 with DUP785. Interestingly, the S1 and S3 increased mtH_2_O_2_ production in the absence of DUP785 when compared to control mitochondria (Fig. 2*B*). Inclusion of DUP785 in reaction mixtures dose dependently decreased the rate of mtH_2_O_2_ production in the absence or presence of S1 and S3 (Fig. 2*B*). Notably, this DUP785 effect was independent of the S1 and S3, which had no significant impact on mtH_2_O_2_ production (Fig. 2*B*). In contrast, leflunomide was significantly less effective than DUP785 in inhibiting mtH_2_O_2_ production (Fig. 2*C*) Indeed, doses of leflunomide as high as 500 μM caused only a small suppression in mtH_2_O_2_ generation in the liver mitochondria energized with dihydroorotate (Fig. 2*C*). Examination of the effect of S1 and S3 alone revealed it had a limited response on mtH_2_O_2_ production (Fig. 2*D*). Furthermore, the administration of the leflunomide with the S1 and S3 also did not induce any significant changes to the production of mtH_2_O_2_ (Fig. 2*D*).

### KMV, VA, and DUP785 suppress OxPhos

**Figure 3:**
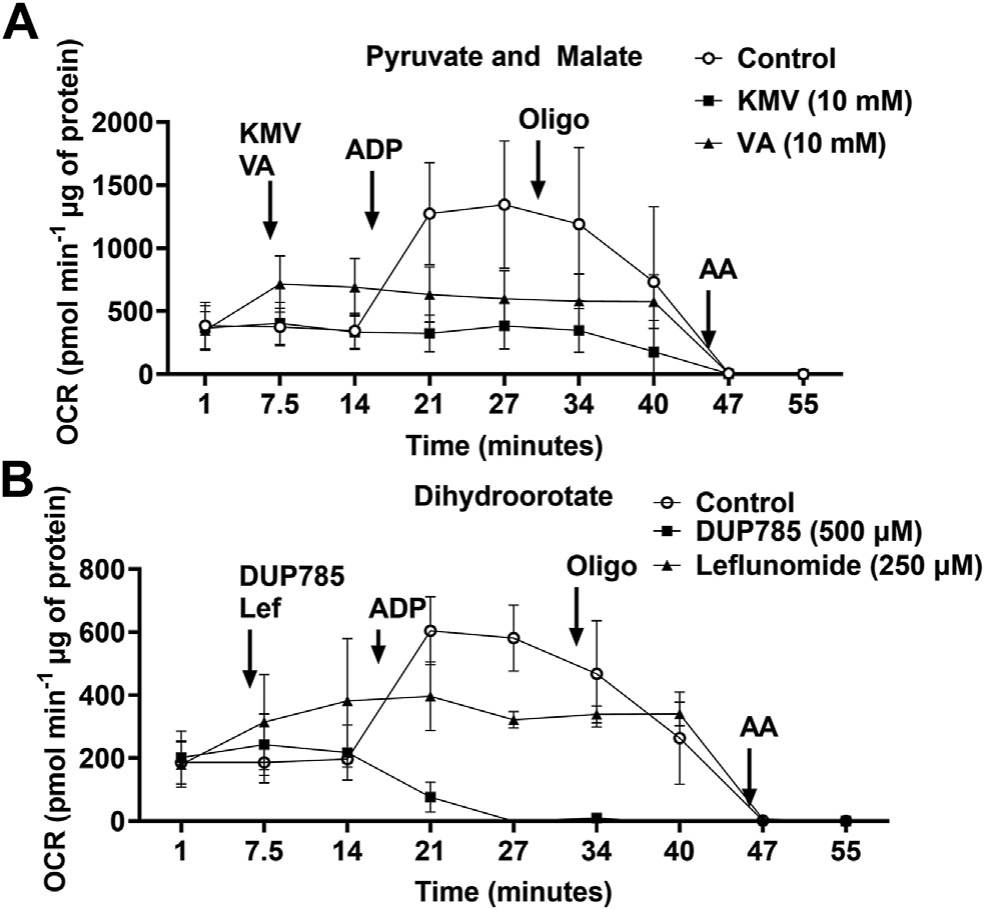
**KMV, valproic acid, DUP785, and leflunomide interfere with OxPhos in isolated liver mitochondria.** Isolated mitochondria were plated into Seahorse TC plates containing respiration buffer supplemented with (*A*) pyruvate and malate or (*B*) dihydroorotate. After assessing state 4 respiration, KMV or valproic acid (*A*) or DUP785 or leflunomide (*B*) were injected into the wells. The impact of all four compounds on state 4, state 3, and state 4_O_ respiration was tested. In the figure panels: N = 4, mean ± SD. AA, antimycin A; KMV, 3-keto-2-methylvaleric acid; Lef, leflunomide; Oligo, oligomycin; OxPhos, oxidative phosphorylation; VA, valproic acid.

Our findings so far are supportive that inhibitors for KGDH and DHODH are potent suppressors for mtH_2_O_2_ production regardless of whether S1 and S3 are added to the reaction mixtures. To verify KMV and VA were blocking KGDH activity in isolated mitochondria, we conducted Seahorse XFe24 analysis on liver mitochondria energized with pyruvate and malate. Injection of KMV resulted in a small increase in state 4 respiration when compared to liver mitochondria treated only with buffer (Fig. 3). Injection of VA did not affect state 4 respiration related to the control (Fig. 3). Treatment with ADP induced a robust increase in state 3 respiration in control liver mitochondria (Fig. 3). By contrast, treatment of mitochondria with KMV or VA prior to stimulating phosphorylating respiration strongly interfered with state 3 OCR (Fig. 3). Complex V in the liver mitochondria treated under control conditions with KMV or with VA responded to the oligomycin treatment, which almost abolished respiration in all the Seahorse wells (Fig. 3). Control mitochondria-oxidizing pyruvate and malate were also responsive to oligomycin, which was evident by the robust inhibition of respiration following the stimulation of state 3 respiration (Fig. 3). However, liver mitochondria treated with KMV or VA displayed diminished responsiveness to oligomycin (Fig. 3). Treatment with ADP revealed dihydroorotate was able to support phosphorylating respiration in liver mitochondria treated with buffer only (Fig. 3). However, the DUP785 treatment completely abolished state 3 respiration when compared to control (Fig. 3). Leflunomide also interfered with state 3 respiration but did not completely inhibit phosphorylating respiration (Fig. 3). Collectively, these findings demonstrate that all four compounds tested in Figure 1 interfere with OxPhos by blocking the activities of KGDH (KMV and VA) and DHODH (DUP785 and leflunomide).

### MitoSNO suppresses mtH_2_O_2_ production in mitochondria-oxidizing pyruvate, but not dihydroorotate

**Figure 4:**
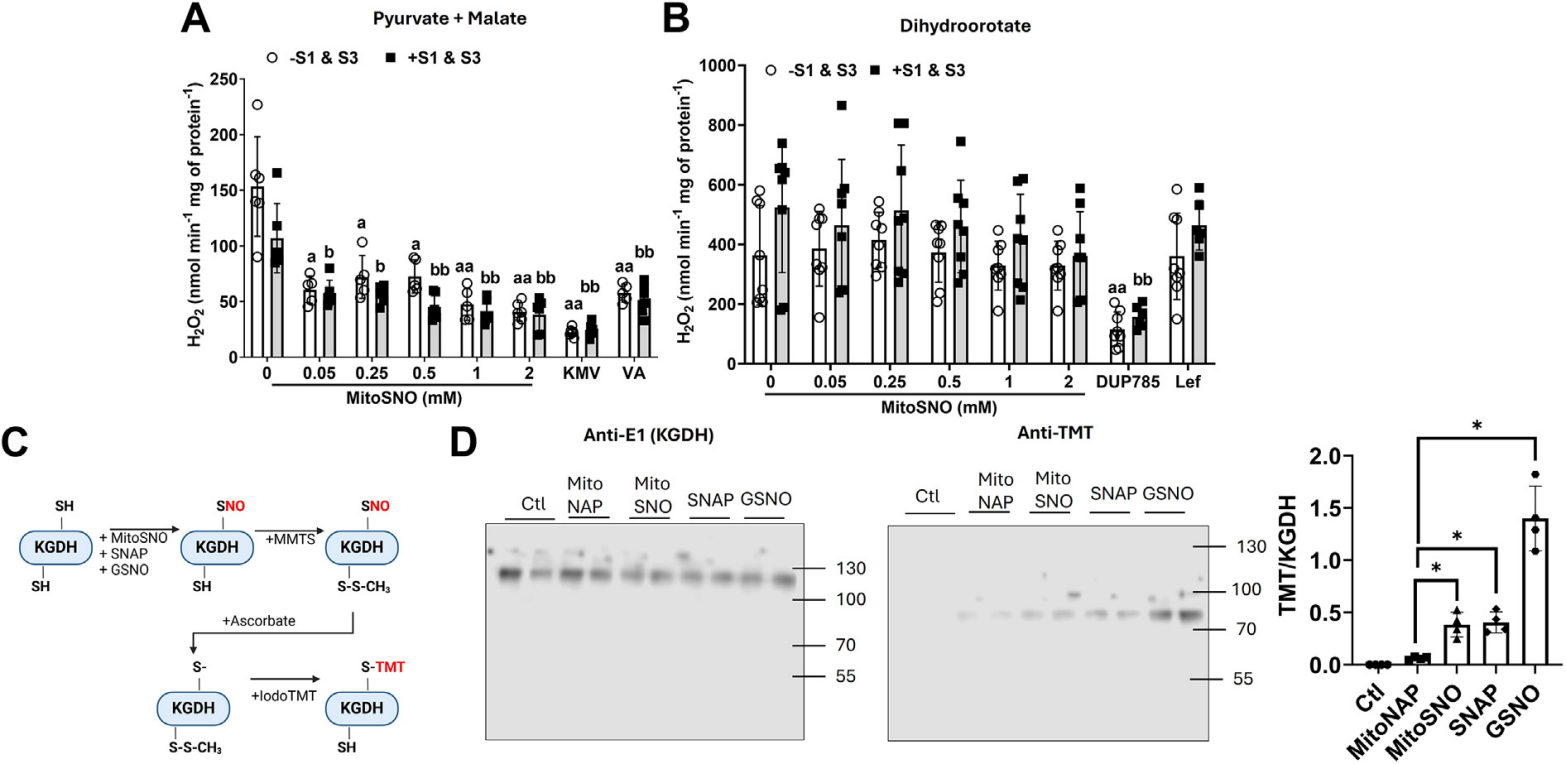
**MitoSNO is just as effective as KMV and valproic acid in the inhibition of mtH_2_O_2_ production by KGDH.***A*, the rate of mtH_2_O_2_ production in liver mitochondria fueled by pyruvate and malate is strongly inhibited by MitoSNO administered in the μM range. Liver mitochondria were given increasing doses of MitoSNO with or without S1 (10 μM) or S3 (10 μM). Treatment of liver mitochondria with KMV (10 mM) or valproic acid (10 mM) and with/without S1 and S3 served as the controls. N = 8, mean ± SD, two-way ANOVA with a Tukey’s *post hoc* test. a = the value is significantly different from the control (0 μM) in the −S1 and S3 group. b = the value is significantly different from the control (0 μM) in the +S1 and S3 group. ◦ (*white bar*) = −S1 and S3 ▪ (*gray bar*) = +S1 and S3. *B*, the rate of mtH_2_O_2_ production in liver mitochondria fueled by dihydroorotate is not affected by MitoSNO administered in the μM range. Liver mitochondria were given increasing doses of MitoSNO with or without S1 (10 μM) or S3 (10 μM). Treatment of liver mitochondria with DUP785 (DUP; 250 μM) or leflunomide (Lef; 250 μM) and with/without S1 and S3 served as the controls. N = 8, mean ± SD, two-way ANOVA with a Tukey’s *post hoc* test. ◦ (*white bar*) = −S1 and S3 ▪ (*gray bar*) = +S1 and S3. *C*, schematic demonstrating the detection of KGDH S-nitrosation using the iodo-TMT switch assay. *D*, KGDH is S-nitrosated by MitoSNO, S-Nitroso-N-acetylpenicillamine (SNAP), or S-nitroso-glutathione (GSNO). Purified KGDH of porcine heart origin was reacted with just buffer alone (control, Ctl, Lanes 1 and 2), MitoNAP (lanes 3 and 4), MitoSNO (lanes 5 and 6), SNAP (lanes 7 and 8), and GSNO (lanes 9 and 10). Blots were quantified using ImageJ software (https://imagej.net/ij/). N = 4. KGDH, α-ketoglutarate dehydrogenase; KMV, 2-keto-3-methylvaleric acid; iodo-TMT, iodoacetyl Tandem Mass Tag; MitoNAP, mitochondria-selective N-acetyl-penicillamine; MitoSNO, mitochondria-targeted S-nitrosating agent; mtH_2_O_2_, mitochondrial hydrogen peroxide.

MitoSNO has emerged as an important tool for investigating how nitro-addition to protein thiols can dynamically modulate mtH_2_O_2_ production in cardiac tissue through complex I regulation (26, 27). Based on this, we reasoned that the MitoSNO compound could also be effective in regulating mtH_2_O_2_ production in a KGDH- and DHODH-dependent manner. Supplementation with increasing amounts of MitoSNO, from 0.05 to 2 mM, revealed a strong dose-dependent inhibitory effect on mtH_2_O_2_ generation by mitochondria-oxidizing pyruvate and malate (Fig. 4*A*). Maximum inhibition with the MitoSNO was achieved at 0.05 mM and the suppression was comparable to reactions supplemented with 10 mM KMV or 10 mM VA, respectively (Fig. 4*A*). Notably, inclusion of 10 μM S1 and S3 had no effect on the rate of mtH_2_O_2_ generation during pyruvate metabolism, regardless of whether MitoSNO, KMV, or VA was in the reaction chambers (Fig. 4*A*).

Next, we examined if MitoSNO could interfere with mtH_2_O_2_ production by DHODH. To our surprise, the MitoSNO treatment had little to no effect on the rate of mtH_2_O_2_ generation, regardless of MitoSNO concentration or if S1 and S3 were included in the reaction chamber (Fig. 4*B*). By comparison, supplementing reactions with 500 μM DUP785 robustly suppressed mtH_2_O_2_ generation by the mitochondria energized with dihydroorotate (Fig. 4*B*). Consistently, leflunomide had no effect on the mtH_2_O_2_ generation by the mitochondria when dihydroorotate was the substrate (Fig. 4*B*). The mammalian DHODH isoforms do not contain any cysteines, and this partly explains why MitoSNO did not inhibit mtH_2_O_2_ production in mitochondria fueled with dihydroorotate (25). However, DHODH injects electrons directly into the ETC which means our findings suggest the MitoSNO does not impact mtROS formed by the respiratory complexes in liver mitochondria.

Together, these data demonstrate KGDH, but not DHODH or the ETC, is a target for MitoSNO-mediated inhibition of mtH_2_O_2_ genesis in liver mitochondria. Thus, we tested if purified KGDH could be S-nitrosated directly by MitoSNO using the iodoacetyl Tandem Mass Tag (iodo-TMT) switch assay and immunoblot analysis. Figure 4*C* illustrates how the iodo-TMT switch assay was conducted. Incubation of the purified KGDH with MitoSNO induced its S-nitrosation (Fig. 4*D*). The S-nitro-modification of KGDH with MitoSNO was compared with well-characterized S-nitrosating agents S-nitroso-N-acetylpenicillamine (SNAP) and S-nitroso-glutathione (GSNO) (Fig. 4*D*). KGDH was modified by SNAP to the same extent as by MitoSNO (Fig. 4*D*). GSNO also induced S-nitrosation and the immunoreactive bands corresponding to the TMT were more intense when compared to MitoSNO and SNAP (Fig. 4*D*). Incubation in mitochondria-selective N-acetyl-penicillamine (MitoNAP) only yielded very faint bands that were immunoreactive toward anti-TMT and exposure to buffer only (control) did not generate an S-nitro–modified KGDH (Fig. 4*D*).

**Figure 5:**
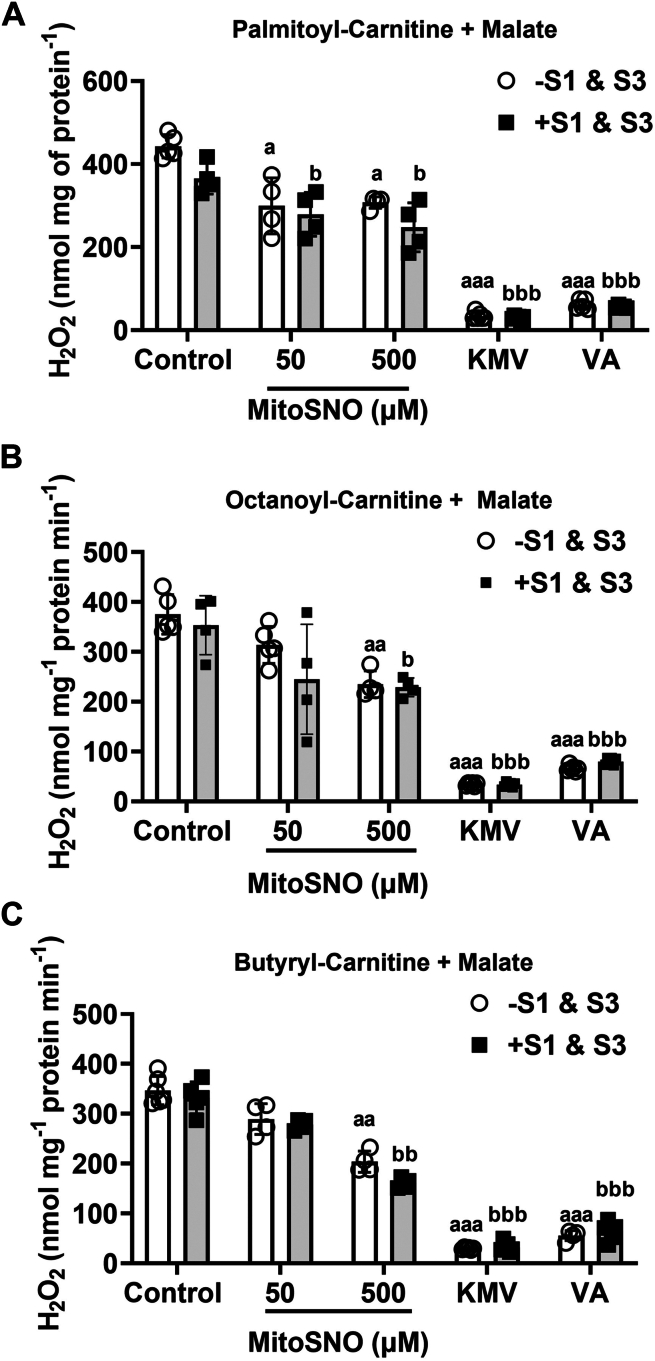
**MitoSNO interferes with mtH_2_O_2_ generation by liver mitochondria fueled with fatty acyl-carnitines and malate.** Liver mitochondria were isolated and treated with/without S1 (10 μM) and S3 (10 μM) and/or 50 or 500 μM MitoSNO. mtH_2_O_2_ production during fatty acid oxidation was measured using a combination of malate with palmitoyl-carnitine (*A*), octanoyl-carnitine (*B*), and butyryl-carnitine (*C*). Reactions were also conducted with liver mitochondria treated with/without S1 (10 μM) and S3 (10 μM) and KMV (10 mM) or valproic acid (10 mM). Malate was included in all reactions. N = 4, mean ± S.D., 2-way ANOVA with a Tukey’s *post hoc* test. a = the value is significantly different from the control (0 μM) in the –S1 & S3 group. b = the value is significantly different from the control (0 μM) in the +S1 and S3 group. ◦ (*white bar*) = −S1 and S3 ▪ (*gray bar*) = +S1 and S3. KMV, 2-keto-3-methylvaleric acid; MitoSNO, mitochondria-targeted S-nitrosating agent; mtH_2_O_2_, mitochondrial hydrogen peroxide; VA, valproic acid.

These experiments were followed up by assessing the impact of MitoSNO on mtH_2_O_2_ generation in mitochondria-oxidizing fatty acylcarnitines with different chain lengths. Our reasoning behind this experiment was based on our recent findings showing KGDH, not complex I and III, is the main mtH_2_O_2_ source during the mitochondrial oxidation of fatty acylcarnitine (13). Inclusion of MitoSNO to reaction chambers at a final concentration of 50 and 500 μM significantly decreased the rate of mtH_2_O_2_ production by liver mitochondria-oxidizing palmitoyl-carnitine when compared to control reactions (Fig. 5). These rates of mtH_2_O_2_ production were not affected by inclusion of S1 and S3 (Fig. 5). By contrast, inclusion of 10 mM KMV almost abolished mtH_2_O_2_ biosynthesis, an effect that was also observed when liver mitochondria were treated with 10 mM VA (Fig. 5). Conducting these experiments with shorter chain fatty acylcarnitines yielded a similar pattern of results. MitoSNO administered in the micromolar range suppressed mtH_2_O_2_ generation when liver mitochondria were supplemented with either octanoyl-carnitine or butyryl-carnitine (Fig. 5). Notably, the 50 μM MitoSNO did not have a significant inhibitory effect when given to liver mitochondria-oxidizing butyryl-carnitine (Fig. 5). Inclusion of S1 and S3 did not significantly alter mtH_2_O_2_ generation and KMV and VA treatment almost abolished production in the mitochondria metabolizing octanoyl- or butyryl-carnitine (Fig. 5).

### Nanomolar MitoSNO suppresses mtH_2_O_2_ production without compromising OxPhos

**Figure 6:**
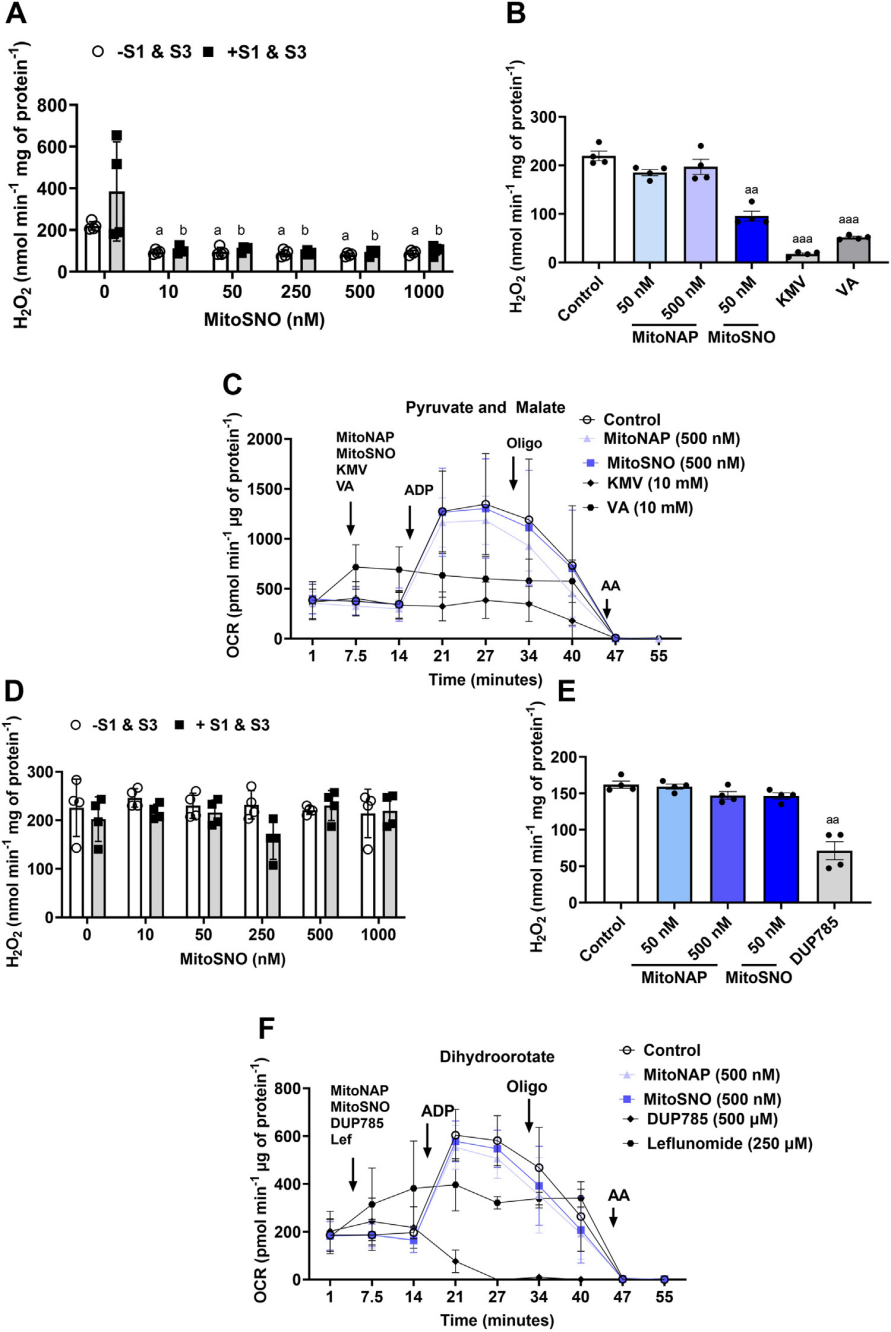
**MitoSNO in the nanomolar range inhibits KGDH-dependent mtH_2_O_2_ production without interfering with OxPhos in isolated liver mitochondria.***A*, the rate of mtH_2_O_2_ production by liver mitochondria fueled with pyruvate and malate and treated with/without S1 (10 μM) and S3 (10 μM) and increasing doses of MitoSNO (from 10 nM to 1000 nM). N = 4, mean ± SD, two-way ANOVA with a Tukey’s *post hoc* test. a = the value is significantly different from the control (0 μM) in the −S1 and S3 group. b = the value is significantly different from the control (0 μM) in the +S1 and S3 group. ◦ (*white bar*) = −S1 and S3 ▪ (*gray bar*) = +S1 and S3. *B*, the MitoNAP precursor compound used to synthesize MitoSNO does not interfere with mtH_2_O_2_ production in liver mitochondria-oxidizing pyruvate and malate. Liver mitochondria were given 50 nM or 500 nM MitoNAP, 50 nM MitoSNO, 10 mM KMV, or 10 mM valproic acid (VA) and then the rate of mtH_2_O_2_ production was measured. N = 4, mean ± SD, one-way ANOVA with a Tukey’s *post hoc* test. a = the value is significantly different from the control. ◦ (*white bar*) = −S1 and S3 ▪ (*gray bar*) = +S1 and S3. *C*, MitoSNO and MitoNAP do not interfere with OxPhos in liver mitochondria-oxidizing pyruvate and malate. Liver mitochondria were plated in Seahorse TC plates and then supplemented with respiration buffer containing pyruvate and malate. After measurement of state 4 respiration, the effect of MitoSNO (500 nM), MitoNAP (500 nM), KMV (10 mM), or valproic acid (10 mM) on the states of respiration were tested. N = 4, mean ± S.D. *D*, the rate of mtH_2_O_2_ production by liver mitochondria fueled with dihydroorotate and treated with/without S1 (10 μM) and S3 (10 μM) and increasing doses of MitoSNO (from 10 nM to 1000 nM). N = 4, mean ± S.D., two-way ANOVA with a Tukey’s *post hoc* test. a = the value is significantly different from the control (0 μM) in the −S1 and S3 group. b = the value is significantly different from the control (0 μM) in the +S1 and S3 group. ◦ (*white bar*) = −S1 and S3 ▪ (*gray bar*) = +S1 and S3. *E*, both the MitoNAP and the MitoSNO do not interfere with mtH_2_O_2_ production in liver mitochondria-oxidizing dihydroorotate. Liver mitochondria were given 50 nM or 500 nM MitoNAP, 50 nM MitoSNO, or 250 μM DUP785 mM and then the rate of mtH_2_O_2_ production was measured. N = 4, mean ± SD, one-way ANOVA with a Tukey’s *post hoc* test. a = the value is significantly different from the control. ◦ (*white bar*) = −S1 and S3 ▪ (*gray bar*) = +S1 and S3. *F*, MitoSNO and MitoNAP do not interfere with OxPhos in liver mitochondria-oxidizing dihydroorotate. Liver mitochondria were plated in Seahorse TC plates and then supplemented with respiration buffer containing pyruvate and malate. After measurement of state 4 respiration, the effect of MitoSNO (500 nM), MitoNAP (500 nM), DUP785 (500 μM), or leflunomide (Lef, 250 μM) on the states of respiration were tested. Oligo, oligomycin; AA, antimycin A. N = 4, mean ± S.D. KGDH, α-ketoglutarate dehydrogenase; KMV, 2-keto-3-methylvaleric acid; MitoNAP, mitochondria-selective N-acetyl-penicillamine; MitoSNO, mitochondria-targeted S-nitrosating agent; mtH_2_O_2_, mitochondrial hydrogen peroxide; OxPhos, oxidative phosphorylation.

Small doses of MitoSNO have been shown to have a cardioprotective effect by inhibiting mtROS generation by complex I (18). Thus, we decided to test if nanomolar amounts of the MitoSNO could also suppress mtH_2_O_2_ generation in a KGDH-specific manner. As shown in Figure 6*A*, MitoSNO had a strong inhibitory effect on mtH_2_O_2_ production in liver mitochondria-oxidizing pyruvate and malate when administered at 10 nM. Concentrations of MitoSNO from 50 to 1000 nM did not result in cause further inhibitory effect (Fig. 6*A*). In addition, the S1 and S3 compounds did not significantly alter mtH_2_O_2_ production when administered with or without the MitoSNO (Fig. 6*A*). Together, these findings show the MitoSNO is an effective inhibitor of KGDH-specific mtH_2_O_2_ generation when administered to liver mitochondria in the nanomolar range. MitoNAP is the thiol-containing lipophilic cation used for MitoSNO synthesis (28). To eliminate the possibility the MitoNAP produced from the denitrosation of the MitoSNO was interfering with the mtH_2_O_2_, we tested its effect on liver mitochondria-oxidizing pyruvate and malate. Inclusion of MitoNAP in reaction chambers at 50 and 500 nM did not significantly alter the rate of mtH_2_O_2_ production related to the liver mitochondria treated with buffer alone (Fig. 6*B*). By contrast, adding MitoSNO to a final concentration of 50 nM or KMV or VA at 10 mM caused robust suppression of mtH_2_O_2_ genesis (Fig. 6*B*). Examination of the impact of MitoSNO on bioenergetics revealed that it does not interfere with the induction of state 3 respiration by ADP when compared to mitochondria respiring under control conditions (*e.g.* treated with buffer only) (Fig. 6*C*). MitoNAP treatment also did not perturb OxPhos (Fig. 6*C*), while KMV and VA treatment both abolished OxPhos (Fig. 6*C*).

Nanomolar MitoSNO did not alter the rate of mtH_2_O_2_ production when liver mitochondria were fueled with dihydroorotate (Fig. 6*D*). MitoNAP also had no effect on the rate of mtH_2_O_2_ production when dihydroorotate was the substrate (Fig. 6*E*). By comparison, DUP785 significantly inhibited mtH_2_O_2_ generation (Fig. 6*E*). S1 and S3 addition to the reaction mixtures also had no effect, consistent with the idea that the ETC is not a significant mtH_2_O_2_ source in liver mitochondria (Fig. 6*D*). Assessment of the impact of MitoSNO on states of respiration in liver mitochondria fueled with dihydroorotate showed it does not interfere with the operation of the ETC (Fig. 6*F*). MitoNAP also did not negatively affect state 3 respiration (Fig. 6*F*). Treatment with DUP785 abolished the response of the liver mitochondria to ADP and leflunomide strongly inhibited OxPhos (Fig. 6*F*).

### MitoSNO prevents the PA- and Fruc-induced inhibition of respiration in Huh-7 cells

**Figure 7:**
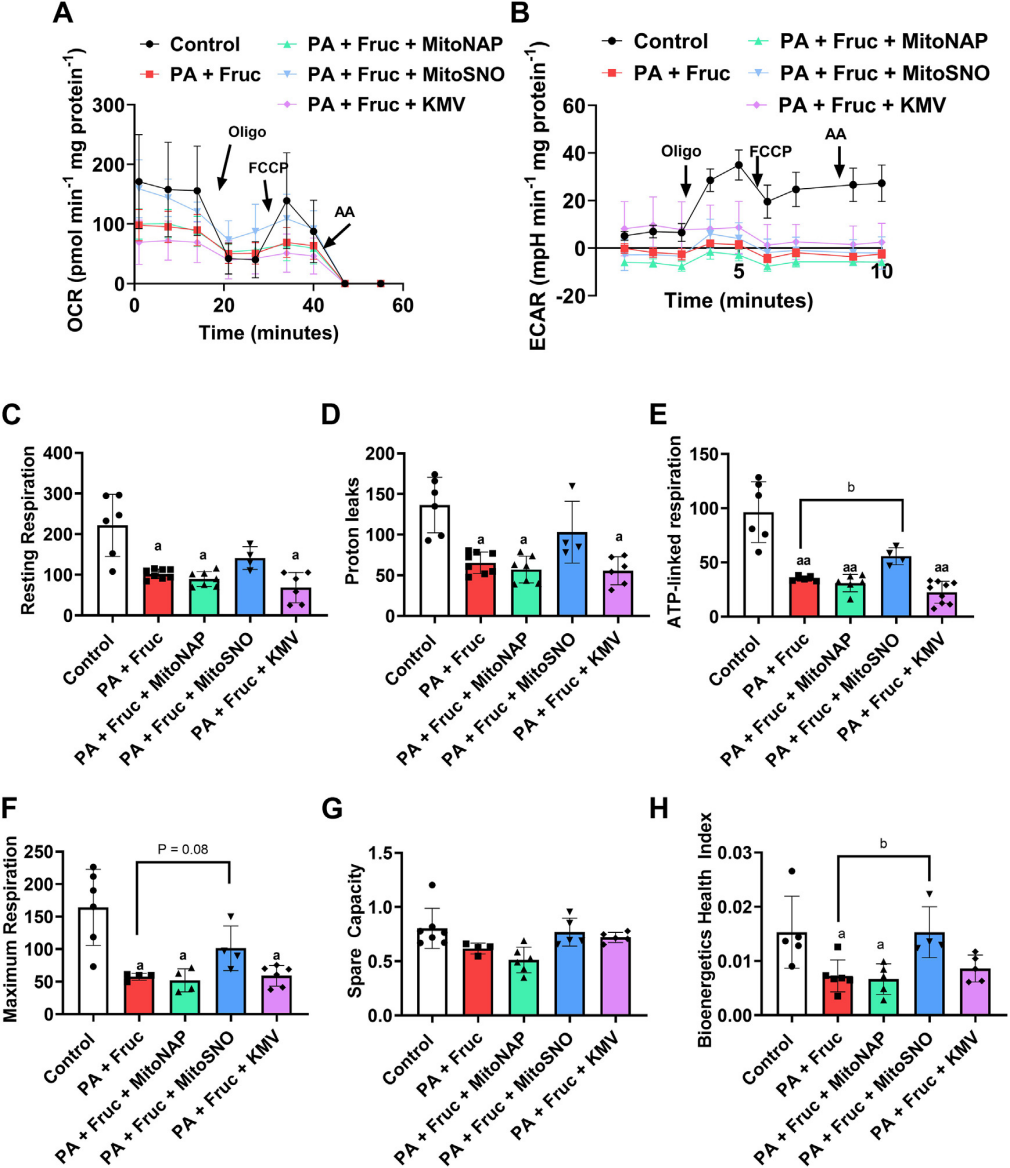
**MitoSNO prevents dysfunctional OxPhos in Huh-7 hepatoma cells overloaded with palmitate and fructose.***A*, representative Seahorse XFe24 trace data for resting oxygen consumption rate (OCR), nonphosphorylating OCR, maximal OCR, and nonmitochondrial O_2_ OCR in Huh-7 hepatoma cells cultured in serum-free control media or serum-free media supplemented with palmitate and fructose with/without MitoNAP (500 nM), MitoSNO (500 nM), or KMV (10 mM). Cells were seeded in a 24-well plate at 50,000 cells/per well, allowed to expand for 24 h, and then treated for 24 h with the conditioned serum-free media. After measuring resting respiration, nonphosphorylating, maximal, and nonmitochondrial OCRs were estimated by treating cells with oligomycin (oligo), FCCP, and antimycin A (AA). N = 4, mean ± SD. *B*, representative Seahorse XFe24 trace data for the extracellular acidification rate (ECAR) in Huh-7 hepatoma cells cultured in serum-free control media or serum-free media supplemented with palmitate and fructose with/without MitoNAP (500 nM), MitoSNO (500 nM), or KMV (10 mM). N = 4, mean ± SD. The results collected in (*A*) were used to calculate the resting (*C*), proton leaks–dependent (*D*), ATP-linked (*E*), and maximal respiration (*F*) and the spare capacity (*G*) and bioenergetics health index (BHI) (*H*) for the Huh-7 cells treated with BSA alone (control) or palmitate and fructose with/without MitoNAP, MitoSNO, or KMV. N = 4, mean ± SD, one-way ANOVA with a *post hoc* Tukey’s test. a = the value is significantly different from the control. b = the value is significantly different from the control. c = the value for the MitoNAP group is significantly different when compared to all other groups. ◦ (*white bar*) = −S1 and S3 ▪ (*gray bar*) = +S1 and S3. BSA, bovine serum albumin; KMV, 2-keto-3-methylvaleric acid; MitoNAP, mitochondria-selective N-acetyl-penicillamine; MitoSNO, mitochondria-targeted S-nitrosating agent; OxPhos, oxidative phosphorylation.

Oxidative distress triggered by the metabolic gridlock-induced overgeneration of mtH_2_O_2_ is a hallmark of hepatic lipotoxicity and the manifestation of NAFLD (29). Based on our findings above, we reasoned that treatment of cultured hepatocytes with MitoSNO would mitigate the lipotoxicity induced by PA and Fruc overloading. Examination of the impact of PA and Fruc on the OCR of the Huh-7 cells revealed that it significantly decreased resting, proton leak, and maximum respiration when compared to cells treated with bovine serum albumin (BSA; control) alone (Fig. 7*A*). Inclusion of MitoSNO at 50 nM in the culture media partially prevented the PA- and Fruc-mediated inhibition of OCR (Fig. 7*A*). The treatment of Huh-7 with PA and Fruc almost abolished extracellular acidification rate (ECAR), a proxy measure for glycolytic flux in cultured cells (Fig. 7*B*). MitoSNO did not improve ECAR in Huh-7 cells overloaded with PA and Fruc (Fig. 7*B*). This observation led us to test if MitoSNO was also impeding the use of glucose for the extracellular acidification of the media. Figure S2 demonstrates treatment of Huh-7 cells with 0.05 mM MitoNAP or MitoSNO does not interfere with OCR or ECAR. However, much higher doses of the MitoSNO (0.5 mM and 1 mM, respectively), abolished both OCR and ECAR (Fig. S2).

Next, using the OCR data from Figure 7*A*, we determined if the MitoSNO treatment preserved resting, proton leak, ATP-linked and maximal respiration and the spare capacity and bioenergetics health index (BHI), key parameters that are used to define mitochondrial (dys)function in cultured cells. PA and Fruc overloading significantly decreased resting and proton leaks–dependent respiration, which could be partially prevented by the MitoSNO treatment (Fig. 7, *C* and *D*). Replication of fatty acid and sugar overloading in the Huh-7 cell culture also compromised ATP-linked respiration and inclusion of MitoSNO mitigated this effect (Fig. 7*E*). Maximum respiration was also rescued by the MitoSNO treatment in Huh-7 cells treated with PA and Fruc (Fig. 7*F*). The spare capacity of the mitochondria in the Huh-7 cells was not affected by the PA and Fruc when compared to BSA-treated cells (Fig. 7*G*). Inclusion of MitoSNO in the media did not alter this mitochondrial function parameter (Fig. 7*G*). PA and Fruc inclusion in culture significantly decreased the BHI, an effect that was prevented by the addition of MitoSNO (Fig. 7*H*). Notably, the MitoNAP and KMV did not rescue resting, proton leak, ATP-linked, and maximal respiration or the spare capacity and BHI in Huh-7 cells loaded with the PA and Fruc.

### MitoSNO mitigates lipotoxicity induced by PA and Fruc overloading by curtailing the overproduction of mtH_2_O_2_

**Figure 8:**
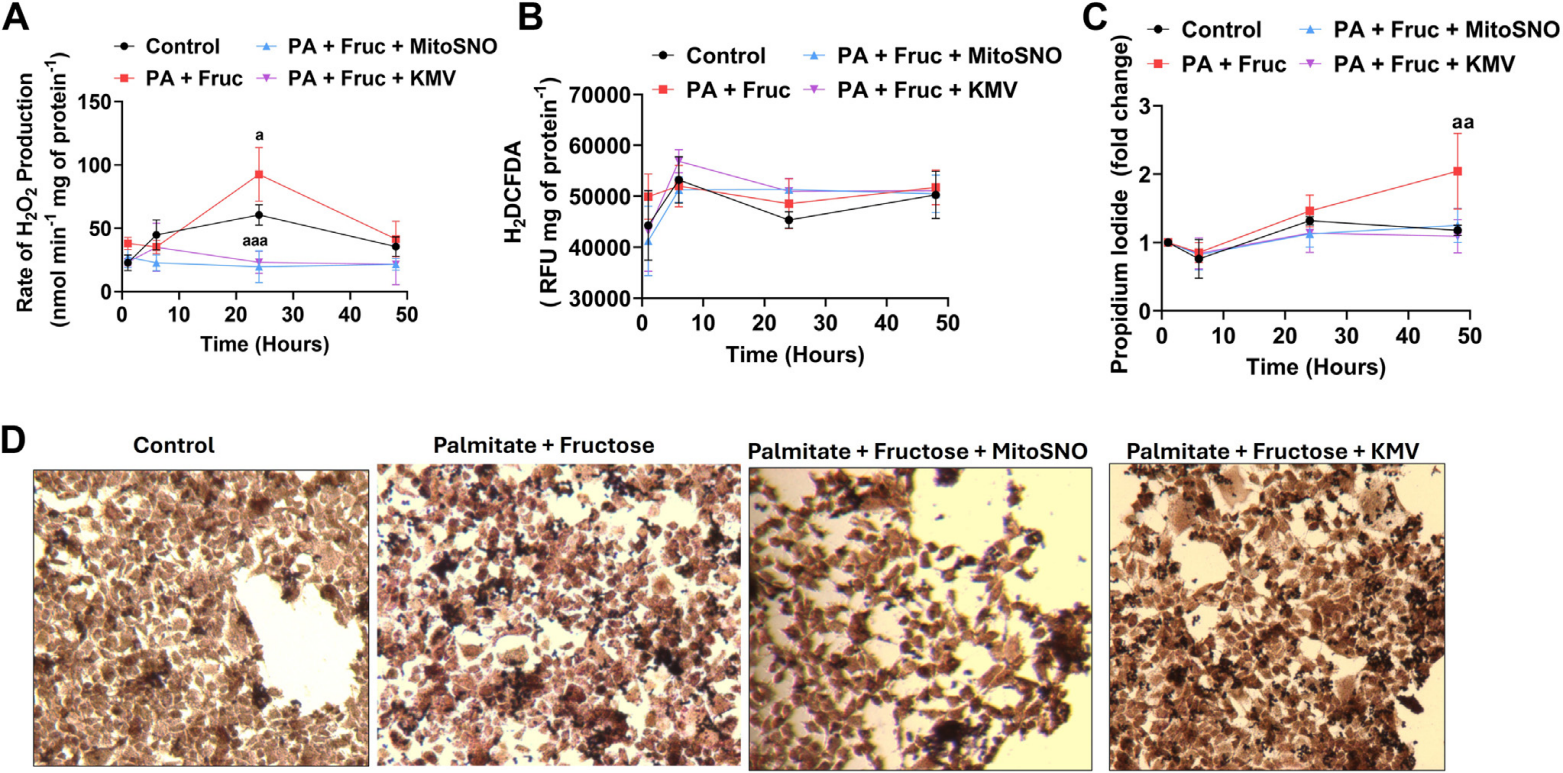
**MitoSNO and KMV mitigate the hyper-generation of H_2_O_2_, cell death, and intrahepatic lipid accumulation in Huh-7 cells exposed to high palmitate and fructose.***A*, assessment of the rate of H_2_O_2_ generation by Huh-7 cells exposed to serum-free media alone (control) or serum-free media supplemented with palmitate and fructose with/without MitoSNO or KMV for 1 h, 6 h, 24 h, and 48 h, respectively. N = 6, mean ± SD. one-way ANOVA with a *post hoc* Tukey’s test. a = values are significantly different when compared to control. *B*, measurement of total oxidative distress using H_2-_DCFH-DA in Huh-7 cells exposed to serum-free media alone (control) or serum-free media supplemented with palmitate and fructose with/without MitoSNO or KMV for 1 h, 6 h, 24 h, and 48 h, respectively. N = 4, mean ± SD, one-way ANOVA with a *post hoc* Tukey’s test. *C*, assessment of cell death in Huh-7 cells by propidium iodide. Cells were exposed to serum-free media alone (control) or serum-free media supplemented with palmitate and fructose with/without MitoSNO or KMV for 1 h, 6 h, 24 h, and 48 h, respectively. N = 8, mean ± SD, one-way ANOVA with a *post hoc* Tukey’s test. a = values are significantly different when compared to control. *D*, visualization of intrahepatic lipid accumulation in Huh-7 cells exposed to serum-free media alone (control) or serum-free media supplemented with palmitate and fructose with/without MitoSNO or KMV for 24 h. Lipid droplets were visualized using Oil Red O. Pictures were taken using a 10x objective. N = 3. DCFDA, dichlorodihydrofluorescein diacetate; KMV, 2-keto-3-methylvaleric acid; H_2_O_2_, hydrogen peroxide; H_2_-MitoSNO, mitochondria-targeted S-nitrosating agent.

As shown in Figure 8*A*, the administration of the PA and Fruc over a 48 h period resulted in the increased production of cellular H_2_O_2_ in cell culture. Notably, the rate of H_2_O_2_ genesis by the Huh-7 treated with PA and Fruc decreased by 48 h but was still statistically higher than cells treated with only BSA (control) (Fig. 8*A*). This increased H_2_O_2_ biosynthesis was completely mitigated by the treatment of cells with KMV over the 48 h period, suggesting the bulk of the H_2_O_2_ genesis caused by the PA and Fruc was from KGDH (Fig. 8*A*). MitoSNO had an effect that was comparable to KMV, indicating the protective effects of MitoSNO are related to the inhibition of KGDH (Fig. 8*A*). Next, we interrogated the effect of the PA and Fruc on the induction of oxidative distress in the Huh-7 cells using dichlorodihydrofluorescein diacetate (H_2_-DCFDA). We detected no differences in H_2_-DCFDA fluorescence between the different treatment groups over the 48 h period (Fig. 8*B*). As H_2_-DCFDA is not an accurate probe for detecting changes in cellular stress, we used additional assays to further elaborate potential cellular protective effects of the MitoSNO. Use of propidium iodide to detect the early induction of apoptosis revealed that PA and Fruc overloading induced a time-dependent increase in cell death (Fig. 8*C*). Indeed, propidium iodide (PI) fluorescence was significantly increased at 24 h of treatment with PA and Fruc, which was increased further at 48 h (Fig. 8*C*). Notably, cotreatment of the PA and Fruc exposed cells with either KMV or MitoSNO completely mitigated this increased PI fluorescence (Fig. 8*C*). Next, using Oil Red O staining, we tested if the MitoSNO and KMV treatments prevented the intrahepatic accumulation of lipids in the Huh-7 cells treated with PA and Fruc. Figure 8*D* demonstrates the PA and Fruc induced a significant accumulation of cytosolic lipids in the Huh-7 cells over a 24 h period. Strikingly, the MitoSNO and KMV treatment mitigated this accumulation as shown by the reduction of Oil Red O staining to that was like the control cells (Fig. 8*D*).

### MitoSNO inhibits the overgeneration of mtH_2_O_2_ and rescues OxPhos in liver mitochondria from male mice fed an HFD

**Figure 9:**
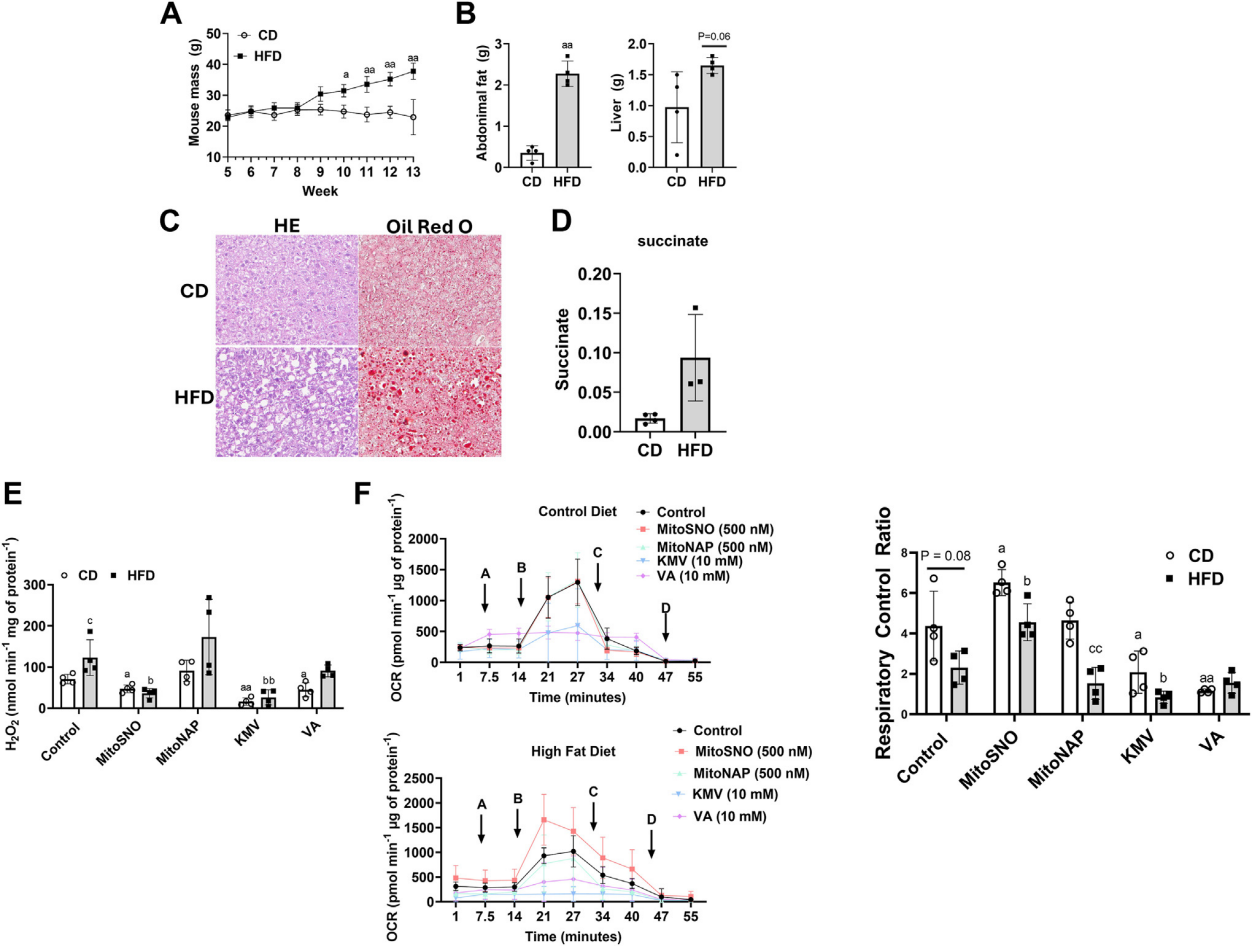
**MitoSNO mitigates the hypergeneration of mtH_2_O_2_ by KGDH and rescues OxPhos in liver mitochondria isolated from mice fed a high-fat diet.***A*, total body mass of male mice fed a control-matched diet (CD; *open circles*) or high-fat diet (HFD; *filled in squares*) from 5 to 13 weeks of age. N = 4, mean ± SD, two-way ANOVA with a *post hoc* Tukey’s test. *B*, abdominal and liver mass of male mice fed a CD or HFD. a = the value is significantly different when HFD is compared to CD at different time points. N = 4, mean ± SD, paired two-tailed Student *t* test. *C*, H&E and Oil *Red* O stains for liver sections collected from male mice fed a CD or HFD. a = the value is significantly different when compared to CD. N = 4. *D*, plasma succinate levels. N = 4, mean ± SD, paired two-tailed Student *t* test. *E*, rate of mtH_2_O_2_ production by liver mitochondria collected from mice fed a CD or HFD. Liver mitochondria were incubated in buffer alone, MitoSNO (500 nM), MitoNAP (500 nM), KMV (10 mM), or valproic acid (VA, 10 mM) prior to measuring mtH_2_O_2_ generation. Pyruvate and malate served as substrates. N = 4, mean ± SD, two-way ANOVA with a *post hoc* Tukey’s test. a = the value is significantly different from the control in the CD group. b = the value is significantly different from the control in the HFD group. c = the value for the MitoNAP group is significantly different when compared to all other groups. ◦ (*white bar*) = −S1 and S3 ▪ (*gray bar*) = +S1 and S3. *F*, measurement of the impact of a CD and HFD on the different states of respiration and the impact of MitoSNO on OxPhos. After assessing state 4 respiration, MitoSNO (500 nM), MitoNAP (500 nM), KMV (10 mM), or valproic acid (VA, 10 mM) were injected into the wells. The impact of all four compounds on state 4, state 3, and state 4_O_ respiration was tested. In the figure panels: *A*: injection of MitoSNO, MitoNAP, KMV, or valproic acid, *B*: injection of ADP, *C*: injection of oligomycin, *D*: injection of antimycin A. Values for state 3 and state 4 (after injection of port A contents) were used to calculate the RCR. N = 4, mean ± SD, 2-way ANOVA with a *post hoc* Tukey’s test. a = the value is significantly different from the control in the CD group. b = the value is significantly different from the control in the HFD group. c = the value for the CD is significantly different from the HFD for individual experimental treatments. ◦ (*white bar*) = −S1 and S3 ▪ (*gray bar*) = +S1 and S3. KGDH, α-ketoglutarate dehydrogenase; KMV, 2-keto-3-methylvaleric acid; MitoNAP, mitochondria-selective N-acetyl-penicillamine; MitoSNO, mitochondria-targeted S-nitrosating agent; mtH_2_O_2_, mitochondrial hydrogen peroxide; OxPhos, oxidative phosphorylation; RCR, respiratory control ratio.

Our findings above indicate MitoSNO could be effective at limiting KGDH-mediated mtH_2_O_2_ over generation by dietary fat overload. To test this, we decided to determine if an acute MitoSNO treatment could limit mtH_2_O_2_ over generation and rescue OxPhos in liver mitochondria from mice fed an HFD for 8 weeks. We chose this model because feeding rodent models an HFD for as short as just 1 week induces oxidative distress leading to the onset of the early stages of NAFLD (17). Mice given the HFD displayed a significant increase in total body mass gain from 8 to 13 weeks of age (Fig. 9*A*). This coincided with an increase in abdominal fat and liver mass (Fig. 9*B*), intrahepatic lipid accumulation (Oil Red O) (Fig. 9*C*), and the vacuolation of the hepatocytes (H&E) (Fig. 9*C*). Plasma succinate, a biomarker for NAFLD, was also higher in the male mice given an HFD for 8 weeks (Fig. 9*D*).

The rate of mtH_2_O_2_ generation was significantly higher in liver mitochondria collected from the mice fed the HFD than samples from the control diet (CD) animals (Fig. 9*E*). MitoSNO suppressed mtH_2_O_2_ generation by the liver mitochondria from mice fed the CD but the effect was far greater in the samples collected from the rodents challenged with the HFD (Fig. 9*E*). Indeed, MitoSNO induced a substantial decrease in mtH_2_O_2_ generation (2-fold) by the mitochondria isolated from the livers of the HFD mice (Fig. 9*E*). The MitoNAP had no significant effect on mtH_2_O_2_ generation (Fig. 9*E*). KMV almost abolished mtH_2_O_2_ production in both CD and HFD liver mitochondria, whereas VA strongly inhibited mtH_2_O_2_ production in CD but not HFD liver mitochondria (Fig. 9*E*). Examination of the acute impact of the MitoSNO on the bioenergetics of the liver mitochondria collected from the CD mice showed it did not significantly affect OxPhos (Fig. 9*F*). Indeed, we observed no changes in state 3 respiratory parameters when CD liver mitochondria were acutely treated with 500 nM MitoSNO (Fig. 9*F*). MitoNAP had no effect on OxPhos on CD liver mitochondria and as expected the short exposure to KMV and VA inhibited oxygen consumption (Fig. 9*F*). Surprisingly, the acute treatment of liver mitochondria from mice fed the HFD with MitoSNO increased state 3 respiration when compared to mitochondria treated with only buffer (control) or MitoNAP (Fig. 9*F*). Calculation of the respiratory control ratios (RCRs) revealed that the acute exposure of liver mitochondria from mice fed the HFD to MitoSNO improved ATP production (Fig. 9*F*). Indeed, the HFD significantly decreased RCR suggesting dietary fat overload does compromise OxPhos (Fig. 9*F*). However, MitoSNO was able to reverse this effect and partially rescue the HFD-induced inhibition of OxPhos (Fig. 9*F*). Surprisingly, the MitoSNO treatment also increased the RCRs in liver mitochondria from mice fed the CD as well (Fig. 9*F*). Together, these findings suggest the MitoSNO compound protects from NAFLD by mitigating the overgeneration of mtH_2_O_2_ and preserving OxPhos.

## Discussion

It is conventionally thought that complexes I and III are the primary sources of mtH_2_O_2_ in mammalian cells. Although this may be true in tissues with a high capacity for OxPhos (*e.g.*, cardiomyocytes or neurons), other flavoproteins in mitochondria are also crucial generators of mtH_2_O_2_ in other cell types (*e.g.*, myocytes, proliferating cells, hepatocytes) (7, 12, 30, 31). KGDH and DHODH are important mtH_2_O_2_ sources that strongly impact cell redox homeodynamics (9, 32, 33, 34). mtH_2_O_2_ production by KGDH is inhibited by the reversible S-glutathionylation or S-nitrosation of the vicinal thiols in the lipoic acid residue of the E2 subunit (35). The redox modification of the E2 subunit was recently shown to protect male C57BL6N mice from NAFLD caused by dietary fat overload by abrogating oxidative distress induced by KGDH (17). By contrast, there is no evidence to suggest DHODH is redox regulated, however, it was recently reported S-glutathionylation catalysts inhibit the production of mtH_2_O_2_ in liver mitochondria fueled with dihydroorotate (36). Together, dynamic redox regulation of mtH_2_O_2_ by KGDH and DHODH may be a therapeutic target for the prevention of hepatic oxidative distress triggered by dietary fat and sugar overload.

In this study, we sought to characterize the mtH_2_O_2_-generating capacity of KGDH, DHODH, complex I and complex III in liver mitochondria and define if mitochondria-selective S-nitrosating agent, MitoSNO, could inhibit this mtH_2_O_2_ generation and protect Huh-7 cells from lipotoxicity caused by PA and Fruc excess. To accomplish this, we used a substrate-inhibitor toolkit consisting of KMV and VA (KGDH inhibitor), DUP785/brequinar and leflunomide (DHODH inhibitor), S1 (complex I inhibitor), and S3 (complex III inhibitor). Our study yielded several novel findings: 1) KGDH and DHODH are potent mtH_2_O_2_ generators, 2) complex I and III produce less mtH_2_O_2_ than KGDH and DHODH, 3) MitoSNO interferes with mtH_2_O_2_ production by KGDH, but not DHODH or the ETC, 4) the MitoSNO mitigates lipotoxicity induced by PA and Fruc overload in Huh-7 hepatoma cells by limiting mtH_2_O_2_ production, 5) low doses of MitoSNO does not interfere with glycolysis or OxPhos and partially rescues mitochondrial respiration during lipotoxicity, 6) KGDH can be directly S-nitrosated by MitoSNO and other S-nitrosation compounds, like SNAP and GSNO, and 7) MitoSNO mitigates mtH_2_O_2_ overproduction and the OxPhos dysfunction caused by an HFD in mice. Together, these findings show MitoSNO administration may be a new pharmacological agent that can be used to alleviate lipotoxicity and NAFLD through the targeted and dynamic S-nitrosation of KGDH.

### KGDH and DHODH are main mtH_2_O_2_ sources in liver mitochondria

In this study, we used S1 and S3 compounds in combination with KGDH and DHODH inhibitors to interrogate the mtH_2_O_2_-generating potential of KGDH, DHODH, complex I and complex III. We chose to focus on these four sources of mtH_2_O_2_ because all four enzymes are consistently found to be important mtH_2_O_2_ generators in normal mammalian cells and cancerous ones (reviewed in (7)). Furthermore, we chose to exclude rotenone (complex I inhibitor) and stigmatellin/antimycin A/myxothiazol (complex III inhibitors) from our toolkit because all these compounds are known to alter electron flow in the ETC, and thereby make our measurements of mtH_2_O_2_ output by all four of these enzymes less accurate. Using S1 and S3 is highly informative as both molecules have been documented to selectively inhibit mtROS production by complex I and III without altering OxPhos, which we have confirmed in this study (3, 4, 5). Furthermore, one main issue related to using the classic ETC inhibitors is these compounds block electron fluxes, which can superficially increase mtH_2_O_2_ generation by flavoproteins upstream from complex I and complex III, like KGDH or DHODH (37, 38). S1 and S3 overcome these issues by abrogating mtROS genesis by complex I and III without interfering with electron conductance to O_2_ in the ETC. We must also consider the limitations associated with using the KGDH and DHODH inhibitors in estimating the rate of mtH_2_O_2_ production as well. KGDH and DHODH inhibitors almost abolished OxPhos meaning these molecules almost completely inhibit electron shuttling to the ETC. This would mitigate mtROS generation by the ETC, potentially underestimating the rate of mtH_2_O_2_ genesis by complexes I and III. However, it must be emphasized S1 and S3 in combination had no effect on mtH_2_O_2_ generation by the liver mitochondria and we have shown previously complex I is a negligible mtROS source in hepatocytes (11).

Using the S1 and S3 compounds in combination with KGDH and DHODH inhibitors, we found both KGDH and DHODH account for a remarkable amount of the mtH_2_O_2_ production by liver mitochondria, whereas the rates of generation in complex I and III were negligible. Although this observation may be surprising, other studies have shown that “unconventional” mtH_2_O_2_ sources can produce equal or even more mtROS than complexes I and III (9, 11, 16). It is important to note that many studies that have measured the mtH_2_O_2_ forming potential of the “unconventional sources” like KGDH and DHODH relied on the use of ETC inhibitors like rotenone or antimycin A, which, as described above, disrupt mitochondrial bioenergetics (9, 10, 11, 39). What sets our study apart from others is we employed the S1 and S3 molecules along with KMV, VA, DUP785, and leflunomide to estimate how much mtH_2_O_2_ is formed by KGDH, DHODH, complex I, and complex III, respectively. By using this approach, we were able to demonstrate KGDH and DHODH, and not complex I and complex III, are key mtH_2_O_2_ generators in liver mitochondria. These findings are consistent with a recent study that implemented S1 and S3 compounds in conjunction with KMV to show KGDH is the main mtH_2_O_2_ source during pyruvate and fatty acid oxidation in liver mitochondria (13). It is crucial to point out that our observations do not discount the importance of complex I and III in the formation of mtROS in other tissues. For example, complex I and III are well known to be potent generators in cardiomyocytes, skeletal muscle, neurological tissue, and several other cell types (39, 40, 41, 42, 43, 44). However, the tissue-, substrate-, and site-specific dependency of mtH_2_O_2_ production should be strongly considered when investigating the mitochondrial sources of mtROS. For instance, Tahara *et al.* showed that mitochondria from liver outpace mitochondria from kidney, brain, heart, and muscle for its capacity to produce mtH_2_O_2_ during phosphorylating respiration (39). Furthermore, Tahara *et al.* found mtH_2_O_2_ generation powered by succinate activates high mtROS production by the ETC in brain, heart, and kidney mitochondria when compared to the liver (39). Additionally, a multitude of studies have delineated the main mtROS sources in cardiomyocytes is complex I and complex III in the ETC (reviewed in (41, 43, 45)). By contrast, several studies have shown liver mitochondria may preferentially produce mtROS from sources upstream from complex I and III, like KGDH (11, 13, 46). It is possible the ETC in hepatic mitochondria produces much less mtROS because they have fewer cristae and liver mitochondria are more poised toward mediating biosynthetic reactions and managing amino acid supply instead of OxPhos than cardiomyocytes, myocytes, and neurons (14, 47, 48, 49). Our finding that S1 and S3 in combination suppress mtH_2_O_2_ in cardiac but not liver mitochondria supports this notion. Thus, the fundamental differences in the arrangement of the mitochondrial metabolic pathways and the ultrastructure of the mitochondrial inner membrane may position KGDH and DHODH to serve as more prominent mtH_2_O_2_ sources in the hepatic liver mitochondria when compared to other tissues.

### mtH_2_O_2_ production by KGDH is inhibited by S-nitrosation by MitoSNO

KGDH is a redox sensor known to be targeted by S-nitrosation and S-glutathionylation of the vicinal lipoic acid thiols on the E2 subunit (20, 50, 51). Targeted S-glutathionylation of KGDH inhibits its activity and impairs mtH_2_O_2_ genesis by the E3 subunit (20, 21). S-nitrosation of the E2 subunit of KGDH also interferes with the NADH and mtH_2_O_2_-producing activities of the enzyme complex (52). We found the MitoSNO compound S-nitrosates KGDH to suppress mtH_2_O_2_ generation in mitochondria of liver and cultured Huh-7 cells. The implementation of MitoSNO to study the role of S-nitrosation in the regulation of KGDH is highly appealing as its lipophilic cation allows it to diffuse through the plasma and mitochondrial membranes and deliver nitric oxide to the matrix. Indeed, the treatment of isolated mitochondria, cultured cells, tissues, and animal models with MitoSNO results in the selective S-nitrosation of the ETC, turning down mtROS production by complex I (18, 28, 53). Recent work has demonstrated S-nitrosation of KGDH and pyruvate dehydrogenase is crucial for the adjustment of the availability of immunomodulatory metabolites that are required for macrophage activation (50, 54, 55). The S-nitrosation of pyruvate dehydrogenase in macrophages was also shown to be key for the regulation of mtH_2_O_2_ generation in proinflammatory signaling (54). Our findings show MitoSNO in the nanomolar range is effective in inhibiting mtH_2_O_2_ generation and that this occurs through the S-nitrosation of KGDH. Importantly, we found the MitoSNO in the nanomolar concentration range did not interfere with OxPhos, whereas the commonly used compounds to site specifically inhibit mtROS production by KGDH (KMV and VA) and DHODH (DUP785 and leflunomide), interfered with mitochondrial bioenergetics. Thus, deploying MitoSNO in studies aimed at identifying the role of S-nitrosation in the regulation of mitochondria-to-cell redox signals can be more informative because it controls the rate of mtH_2_O_2_ without affecting mitochondrial fuel metabolism and OxPhos.

### MitoSNO has the therapeutic potential to mitigate the manifestation of NAFLD

There is considerable therapeutic interest in the targeted redox modification of mitochondrial proteins as it protects from the irreversible oxidation of protein thiols and can restore cell redox homeodynamics (56, 57). MitoSNO is a mitochondria-targeted nitric oxide donor that was developed to study the protective effects of S-nitrosation reactions (28). Selective mitochondrial S-nitrosation is achieved using a triphenylphosphonium ion that promotes the membrane potential-dependent matrix accumulation of the MitoSNO by several 100-fold (28). MitoSNO was found to be effective at vasodilation but has mostly been investigated in the context of preventing ischemia-reperfusion injury to cardiac, brain, and skeletal muscle tissue and postmyocardial infarction heart failure (18, 19, 26, 28, 53, 58). Specifically, the MitoSNO elicits these protective effects through the S-nitrosation of Cys^39^ in the ND3 subunit of complex I, which nullifies mtROS overgeneration caused by reverse electron flow from succinate during tissue reperfusion (18). The MitoSNO has also been found to protect the irreversible oxidative deactivation of the ETC by selectively modifying thiols in complexes II, III, IV, and V (53). This suggests the MitoSNO exerts its protective effects through the reversible modification of multiple proteinaceous cysteine residues in mitochondria.

The therapeutic potential of MitoSNO also applies to NAFLD, a disease that manifests because of mitochondrial metabolic gridlock and the induction of oxidative distress triggered by excess dietary fat and sugar consumption. Mitochondria-selective antioxidants like anti-OxCIN_4_, MitoQ, mito-vitamin E, or MitoTEMPO mitigate NAFLD by abating lipotoxicity, inflammation, hepatic ballooning, and fibrosis caused by feeding murine models a Western-style or HFDs (59, 60, 61). Two recent investigations conducted by our group found the source of the mtH_2_O_2_ that triggers oxidative distress caused by dietary fat overload is KGDH (13, 17). Notably, the induction of KGDH S-glutathionylation by the ablation of the *glutaredoxin-2* gene (*Glrx2*), which inhibits mtH_2_O_2_ generation by the enzyme complex, abrogates the oxidative distress induced by the HFD, preventing plasma succinate accumulation, intrahepatic lipid accumulation, fibrosis, and hepatic ballooning (17). Here, we show the MitoSNO prevents lipotoxicity in cultured Huh-7 cells through the inhibition of mtH_2_O_2_ generation by KGDH. The combination of the PA and Fruc increased Huh-7 intracellular lipid accumulation and cell death. This was associated with the disruption of cellular bioenergetics and the overgeneration of H_2_O_2_. Inclusion of the MitoSNO compound in culture media supplemented with PA and Fruc partially recovered OxPhos and abrogated intracellular lipid accumulation, cell death, and the overgenesis of cellular H_2_O_2_. Crucially, the KMV elicited some of the same effects as MitoSNO which include prevention of lipid accumulation, cell death and the high rate of H_2_O_2_ production. Although the KMV did not rescue OxPhos, this demonstrates the protective effects of the MitoSNO could be mediated through KGDH. The capacity of MitoSNO to mitigate mtROS overproduction and rescue OxPhos was recapitulated in liver mitochondria collected from mice fed an HFD. Feeding mice an HFD caused increased hepatic mass, abdominal fat hypertrophy, intrahepatic lipid accumulation, and higher succinate concentration in the blood, which coincided with the overgeneration of mtH_2_O_2_ and decreased OxPhos. The overproduction of mtH_2_O_2_ and diminished OxPhos capacity were reversed by an acute incubation with MitoSNO. KMV and VA also prevented the over production of mtH_2_O_2_ by the liver mitochondria isolated from the mice fed an HFD, but both compounds strongly interfered with mitochondrial respiration. When taken together, MitoSNO has the potential of being an effective agent for the mitigation of NAFLD manifestation through dynamic regulation of KGDH.

## Experimental procedures

### Chemicals

Mitochondria-selective S-nitrosating agent, MitoSNO, and its precursor, mitochondria-selective N-penicillamine (MitoNAP), was synthesized and verified by Professor Mike Murphy’s group as described in (28). Acrylamide solution, ammonium persulfate, Laemmli buffer, and nitrocellulose membrane were obtained from Bio-Rad. Goat anti-mouse-horseradish peroxidase (HRP)-conjugated secondary antibody, goat antirabbit-HRP conjugate secondary antibody, KGDH E1 subunit primary antibody, and the succinate detection kit were obtained from Abcam. Chemiluminescence detection agent, H_2_-DCFDA, PI, 3-(4,5-dimethylthiazol-2-yl)-5-(3-carboxymethoxyphenyl)-2-(4-sulfophenyl)-2H-tetrazolium assay kit, Amplex UltraRed (AUR) and Pierce S-nitrosation kit were obtained from Thermo Fisher Scientific. ADP, S1QEL 1.1 (S1), S3QEL 2 (S3), carbonyl cyanide 4-(trifluoromethoxy)phenylhydrazone, antimycin A, VA, DUP785, leflunomide, purified KGDH of porcine heart origin, BSA, EGTA, KMV, glycine, Hepes, HRP, malic acid, mannitol, MgCl_2_, NaCl, NADH, oligomycin, butyryl-carnitine, octanoyl-carnitine, palmitoyl-carnitine, ponceau S, pyruvic acid, superoxide dismutase (SOD), sodium azide, dihydroorotate, and sucrose were all obtained from Millipore Sigma.

### Animal care and mitochondrial isolations

Animals were cared for in accordance with the principles and guidelines of the Canadian Council on Animal Care and the Institute of Laboratory Animal Resources. Animal experiments were approved by the Facilities Animal Care Committee in the Faculty of Agricultural and Environmental Sciences at McGill University. Male C57BL6N mice were purchased from Charles River at 9 to 10 weeks and were maintained standard maintenance diet (Inotiv TD2020X) at 21 °C on a 12-h light/dark cycle until 11 to 12 weeks of age. For experiments where mice were fed to an HFD, male C57BL6N mice were purchased from Charles River at 4 weeks of age, fed the TD2020X diet for 1 week and then placed on a were placed on an HFD (Inotiv TD.06415) or matched low-fat CD (Inotiv TD.06416) until 12 weeks of age. Mouse body mass and blood glucose were measured weekly. All mice were euthanized by cervical dislocation after being heavily anesthetized with isoflurane. Blood samples were collected by cardiac puncture from the mice fed the CD or HFD using heparinized needles. Plasma samples were stored at −80 °C for analysis of succinate levels using the Abcam Succinate Detection Kit (assays were conducted according to the manufacturer's instructions). A small piece of liver was kept for histological analysis of cell morphology by HE and intrahepatic lipid levels using Oil Red O. Liver samples were prepared for histology as described previously and the staining and imaging was performed by the McGill University Histology Core (17).

Mitochondrial isolations were conducted on surgically removed livers placed in either ice-cold MESH buffer (200 mM mannitol, 1 mM EGTA, 70 mM sucrose, 20 mM Hepes, pH 7.4) or ice-cold MESH-S buffer (220 mM, 1 mM EGTA, 70 mM sucrose, 10 mM Hepes, 10 mM pyruvate, and 2 mM malate, pH 7.2; MESH-S). The MESH-S buffer is required for Seahorse XFe24 assays. Mouse livers were cut into pieces, washed in MESH or MESH-S, and minced using a razor on a Teflon watch glass on ice. Samples were homogenized in 25 ml of MESH or MESH-S supplemented with defatted BSA using Teflon pestles and a Glas-Col variable speed homogenizer. Homogenates were centrifuged at 900*g* and 4 °C for 9 min to pellet cellular debris and nuclei (Sorvall Lynx 4000). The supernatant was collected and centrifuged at 12,000*g* at 4 °C for 9 min to pellet the mitochondria. The pellet was washed and resuspended in 500 μl of MESH or MESH-S and stored on ice for assays. Protein equivalents were determined using a Bradford assay. BSA was used to construct the calibration curve.

### Cell culture

The Huh-7 human hepatoma cell line was provided to use by the Professor Kostas Pantopoulos (McGill University, Lady Davis Institute for Medical Research, Jewish General Hospital). The Huh-7 cells were maintained and expanded in complete Dulbecco’s modified Eagle medium (DMEM; Wisent, Cat # 319-005CL) containing 10% (v/v) fetal bovine serum (Wisent), and 1% (v/v) penicillin/streptomycin (Gibco). Media was changed every 2 days and cells were passaged when they reached ∼70% confluency. For Seahorse experiments, cell density for measures of OCR and ECAR were optimized by plating 5000 cells to 50,000 cells. The optimal cell density for assays was determined to be 50,000 cells. Cell densities were also optimized for the AUR, 3-(4,5-dimethylthiazol-2-yl)-5-(3-carboxymethoxyphenyl)-2-(4-sulfophenyl)-2H-tetrazolium, PI, and H_2_-DCFDA assays (optimal seeding density was determined to be 10,000 in 96-well plate cells). For lipotoxicity assays, the Huh-7 cells were treated with either serum-free DMEM or Krebs Ringer buffer (KRB; recipe containing 5 mM glucose and 1 mM glutamine) supplemented with PA complexes to BSA at a 1:1 ratio (final concentration of 0.1 mM) and 1 mM Fruc. The PA–BSA complex was concocted as described in (62). To determine if MitoSNO or KMV protected from lipotoxicity, the serum-free DMEM or KRB were containing PA and Fruc was supplemented with MitoSNO (500 nM), MitoNAP (500 nM), or KMV (10 mM). Cultures supplemented with BSA only served as the control.

### AUR assays

Isolated mitochondria were diluted to 5 mg/ml in MESH and stored on ice prior to running the assays. Samples were then diluted to a final concentration of 0.5 mg/ml in MESH in the wells of a 96-well black plate and incubated for 5 min at 25 °C. MitoSNO (0.05–2 mM or 10–1000 nM), MitoNAP (50 or 500 nM), KMV (10 mM), VA (0–25 mM), DUP785 ((0–250 μM)), or leflunomide (0–100 μM) were added to the reactions with or without S1 or S3 (10 μM each), followed by a 15-min incubation at 25 °C. HRP, SOD, and AUR were each added to final concentrations of 3 U/ml, 25 U/ml, and 20 μM, respectively, in each well. Substrates were then added. Reactions were initiated by the addition of 10 mM pyruvate and 2 mM malate, 100 μM butyryl-carnitine, octanoyl-carnitine, or palmitoyl-carnitine, and 2 mM malate or 1 mM dihydroorotate. For cell-based assays, the Huh-7 were seeded in 96-well plates and allowed to grow for 24 h. Cells were then treated for 1 h, 6 h, 24 h, and 48 h with KRB containing PA-BSA and Fruc or BSA alone or PA-BSA and Fruc with MitoSNO or KMV as described above. Cultures were then supplemented with KRB alone containing HRP, SOD, and AUR to measure H_2_O_2_ generation. For all assays with isolated mitochondrial and cells, the conversion of AUR to fluorescent resorufin was measured every 30 s to 1 min for 1 to 20 min at 565/610 nm and 37 °C using a Cytation 5 microplate reader controlled by Gen 5 3.11 software (https://www.agilent.com/en/support/biotek-software-releases). mtH_2_O_2_ production was normalized to mitochondrial protein equivalents and background fluorescence. The rate of mtH_2_O_2_ production was calculated using a linear calibration curve constructed using 1 to 1000 nM H_2_O_2_ and the AUR reagents.

### Seahorse XFe24 assays

XFe24 assays with isolated mitochondria were performed as described in (13). Samples were diluted to 0.2 mg/ml in MESH-S and 50 μl of the preparation was added to the wells of a 24-well XFe24 tissue culture plate. The plate was centrifuged at 1200×*g* for 20 min in a Sorvall X Pro Series swing bucket centrifuge at room temperature. Four hundred fifty microliters of respiration buffer (MESH supplemented with 10 mM KH_2_PO_4_, 2 mM MgCl_2_, 0.1% (w/v) defatted BSA, 5 mM pyruvate, and 2 mM malate or 1 mM dihydroorotate, respectively) and then incubated for 30 min at 37 °C. The bioenergetics of the liver mitochondria was interrogated by first measuring state 4 rate for oxygen consumption (OCR, respiration in the presence of the substrate only). For all assays, KMV (10 mM), VA (10 mM), DUP785 (500 μM), leflunomide (250 μM), MitoSNO (500 nM), or MitoNAP (500 nM) were then injected into the reaction chambers and their impact on state 4 respiration measured. State 3 OCR was then examined by injecting ADP (4 mM final concentration) into the wells, followed by the measurement of state 4_O_ (injection of oligomycin; 2.5 μg/ml final concentration). Antimycin A (4 μM) was then injected to estimate the OCR that was not associated with ETC function. OCR values were corrected for the background O_2_ consumption determined from the respiration rate after antimycin A was added. All values were normalized to the protein equivalents to mitochondria per well.

Analysis of OCR and ECAR in Huh-7 cells was conducted by first seeding and growing the cells in an XFe24 tissue culture plate. Cells were grown for 24 h and then treated with serum-free DMEM supplemented with PA and Fruc and MitoSNO, MitoNAP, or KMV as described above. After a 24-h exposure to these culture conditions, the Huh-7 cells were washed twice and supplemented with 500 μl Seahorse respiration buffer (HCO_3_^-^ and phenol red free DMEM containing 5 mM glucose, 1 mM glutamine, and 1 mM pyruvate, pH 7.4). After a 30 min at 37 °C in the Seahorse respiration buffer, cell bioenergetics were interrogated by first measuring basal OCR and ECAR. This was followed by the injection of oligomycin (2.5 μg/ml), carbonyl cyanide 4-(trifluoromethoxy)phenylhydrazone (4 μM), and antimycin A (4 μM) to measure nonphosphorylating respiration, maximal respiration, and nonmitochondrial respiration. Basal, nonphosphorylating, and maximal OCR values were normalized to nonmitochondrial respiration. ATP-linked and proton leak–dependent respiration, the reserve capacity of mitochondria, the apparent state (State_app_) of respiration, and the BHI of the mitochondria were calculated using normalized basal, nonphosphorylating, and maximal OCR values as described in (63). Results were normalized to protein content per well and for all assays, injection protocols were developed using Wave Controller Software 2.4 (https://www.agilent.com/en/product/cell-analysis/real-time-cell-metabolic-analysis/xf-software/seahorse-wave-controller-software-2-4-2-740903).

### Huh-7 cell death, oxidative distress, and lipid accumulation assays

Huh-7 cells were seeded in 96-well plates and allowed to grow for 24 h. Cells were then treated for 1 h, 6 h, 24 h, and 48 h with KRB containing PA-BSA and Fruc or BSA alone or PA-BSA and Fruc with MitoSNO or KMV as described above. At the various time intervals, the KRB was removed and replaced with fresh KRB containing PI (10 μM) or H_2_-DCFDA (10 μM) and then incubated for 30 min at 25 °C. The KRB was then removed, cells washed twice, and then the fluorescence was read at excitation and emission wavelengths of 535 nm and 617 nm (PI) and 485 nm and 535 nm (H_2_-DCFDA). Intrahepatic lipid accumulation was conducted on Huh-7 cells grown to ∼70% in a 6-well plate. Cells were fixed and stained with Oil Red O solution as described in (64). Cells were visualized with bright field using a Leica DM microscope and a 10x objective.

### TMT switch assays

Our ability to determine if KGDH was S-nitrosated was conducted using the TMT switch assay. All assay steps were performed according to the manufacturer’s instructions (Thermo Fisher Scientific; PI90105). Briefly, purified KGDH of porcine heart origin (MilliporeSigma) was diluted in the HENS buffer supplied in the kit to 1 mg/ml. The solution was vortexed vigorously. Next, MitoNAP, MitoSNO, SNAP, or GSNO were added to individual reactions at a final concentration of 500 nM (MitoNAP and MitoSNO) and 250 μM (SNAP and GSNO). Samples were then incubated for 15 min at 37 °C. Excess reactant was then removed using Zeba size exclusion desalting columns. Methyl methanethiosulfonate was then added to block any free thiols in the mixture. After removing the excess methyl methanethiosulfonate with desalting columns, the S-nitroso groups on KGDH were reduced with ascorbate and the free thiols modified with iodoacetyl-TMT. Samples were then treated with Laemmli buffer and 20 μg of sample was electrophoresed in an isocratic 10% SDS-denaturing gel. Proteins were transferred to nitrocellulose membranes, blocked, and then probed overnight at 4 °C with either primary anti-KGDH antibody (Abcam; ab137773) or anti-TMT antibody (Thermo Fisher Scientific; PI90105). Membranes were then washed, probed with HRP-conjugated goat anti-rabbit secondary antibody and then visualized using chemiluminescence and a Li-Cor C-Digit Scanner.

### Statistical analyses

All data calculations were conducted in Microsoft Excel and results collated into GraphPad Prism 9 (https://www.graphpad.com/updates/prism-900-release-notes) for statistical analysis. Results were analyzed using one-way and two-way ANOVAs with a Tukey’s *post hoc* test or a paired, two-tailed Student *t* test. a, b, c = *p* ≤ 0.05, aa, bb,cc = *p* ≤ 0.01, aaa, bbb, ccc = *p* ≤ 0.005, aaaa, bbbb, cccc = *p* ≤ 0.001.

## Conclusion

In conclusion, we identified KGDH as one of the main sources of hepatic mtH_2_O_2_ by deploying complex I and III mtROS inhibitors that do not disrupt OxPhos in conjunction with chemical deactivators for KGDH. This approach also led to the identification of KGDH as the main site for MitoSNO-mediated inhibition of hepatic mtH_2_O_2_ production, which led us to find the S-nitrosation of KGDH prevents lipotoxicity in cultured liver cells and prevents the overgeneration of mtROS by liver mitochondria isolated from male mice fed an HFD. Collectively, these findings have strong implications for understanding the source of oxidative distress in the manifestation of NAFLD and how mitochondria-targeted compounds like MitoSNO could be implemented to mitigate the progression of this disease. KGDH is a mitochondrial redox sensor that serves as both a source and sink for mtH_2_O_2_ (38, 65, 66). Covalent redox modifications like S-nitrosation serve as a negative feedback loop to regulate the rate of mtH_2_O_2_ production by KGDH through the reversible modification of the E2 subunit (35). Our findings show for the first time that targeted redox regulation of KGDH with the MitoSNO compound has the therapeutic potential for preventing hepatic lipotoxicity in response to excessive fat and sugar consumption.

## Limitations of the study

We used isolated liver mitochondria from C57BL6N mice and cultured Huh-7 cells to identify the main sources of hepatic mtH_2_O_2_ generation and define the protective effects of MitoSNO against the overgeneration of mtH_2_O_2_ by KGDH. A major limitation to this study is we did not examine sex as a biological modifier for mtH_2_O_2_ production. This study was executed using male C57BL6N mice only and Huh-7 cells, which are of human male origin. Although this is a limitation here, it is important to point out that our group previously showed sex is a modifier of mtH_2_O_2_ (13, 17, 52). In these studies, we provide evidence demonstrating KGDH is a main source of mtH_2_O_2_ production in liver mitochondria from male and female mice. In our hands, we showed the female mice had an innate resistance toward development of NAFLD caused by dietary fat overload because of the greater mtROS handling and a more efficient OxPhos system. However, further studies are needed to determine if sex also modifies the response to MitoSNO. Another main limitation to this study is we did not test if MitoSNO directly prevents NAFLD in our rodent models. Here, male mice were administered a CD and HFD and then hepatic mitochondria were isolated to determine if MitoSNO could mitigate the overgeneration of mtH_2_O_2_ caused by the intake of the high fat chow. Thus, future studies will strongly consider the use of animal models where the MitoSNO compound is administered directly to mice with the HFD to define if KGDH S-nitrosation can mitigate NAFLD induced by dietary fat overload. Last, although the S1 and S3 compounds have been shown by several groups to selectively limit mtROS formation by complex I and III without disrupting OxPhos, the mechanism underlying how these molecules do so still need to be delineated.

## Data availability

Data are available in the main text or the Supporting information.

## Supporting information

This article contains supporting information.

## Conflict of interest

The authors declare that they have no conflicts of interest with the contents of this article.

## Supporting information

Supplemental JBC Chalifoux.docx
