# Supplementary material for: MitoSNO inhibits mitochondrial hydrogen peroxide generation by α-ketoglutarate dehydrogenase
Source: J Biol Chem. 2025 Apr 16;301(6):108510. doi: 10.1016/j.jbc.2025.108510 (PMC12144464; doi:10.1016/j.jbc.2025.108510)
Supplement: Supplemental JBC Chalifoux.docx [file mmc1.docx]

**Figure S1**


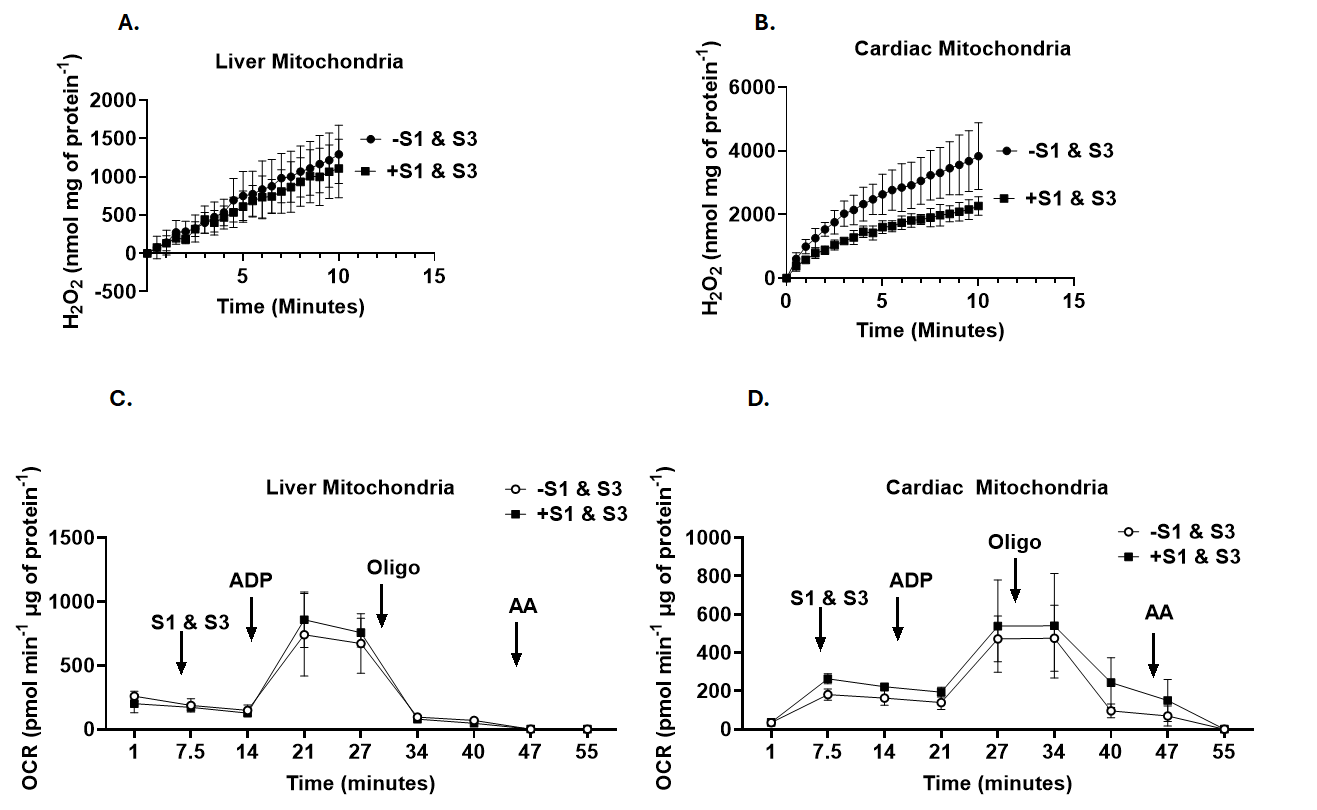


**Figure S1**: Assessment of the impact of the combination of S1 (10 µM) and S3 (10 µM) on the production of mtH_2_O_2_ and OxPhos in liver mitochondria (**A, B**) and cardiac mitochondria (**C**, **D**) isolated from C57BL6N mice. N=4, mean±SD.


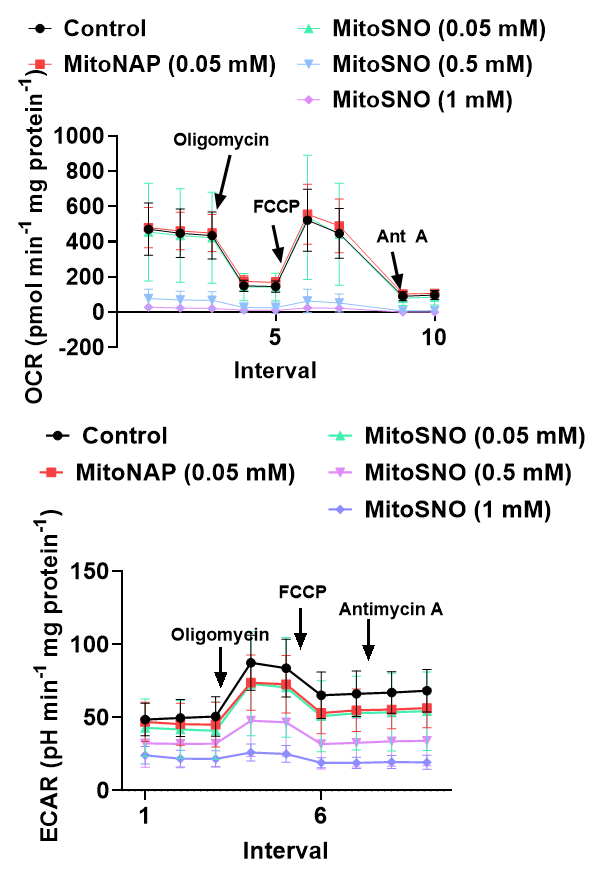


**Figure S2**: MitoSNO in the low micromolar range does not interfere with OxPhos or glycolysis in Huh-7 hepatoma cells. The Huh-7 cells were seeded at 25,000 cells/mL, grown for 24h, and then treated with serum-free media containing MitoNAP or MitoSNO. Non-phosphorylating, maximal, and non-mitochondrial oxygen consumption rates (OCR) were measured following the sequential injection of oligomycin, FCCP, and antimycin A (Ant A). N=4, mean±S.D.
